# How Does Peripheral Vision Feel Rich? A Selective Review

**DOI:** 10.3390/jemr19050104

**Published:** 2026-09-18

**Authors:** Amelia Beatson, Anna Metzger, Matteo Toscani

**Affiliations:** School of Psychology, Bournemouth University, Poole BH12 5BB, UKametzger@bournemouth.ac.uk (A.M.)

**Keywords:** peripheral vision, active vision, spatial vision

## Abstract

Human vision is profoundly non-uniform. Spatial resolution, contrast sensitivity, colour discrimination decrease, and the appearance of visual features become increasingly distorted with retinal eccentricity, yet visual experience appears remarkably rich and stable across the entire visual field. This apparent paradox has been a central challenge in vision science: how can a perceptually rich world emerge from a sensory system that samples only a small fraction of the environment with high precision? We argue that cognitive biases alone may be insufficient to explain the apparent richness of peripheral vision and review evidence suggesting that perceptual processes may also contribute. Information sampled in central vision can be extrapolated to peripheral appearance, while prior knowledge and learned regularities allow the visual system to infer missing or distorted peripheral information. These processes operate within the constraints of active vision, where eye movements selectively sample task-relevant information rather than building a complete detailed representation of the scene. We propose that the interaction between centre-to-periphery extrapolation and learned predictions may contribute to rich peripheral experience, specially where we direct attention, allowing the visual system to maintain a coherent and useful representation of the world.

## 1. The Paradox of Peripheral Vision

**Figure 1:**
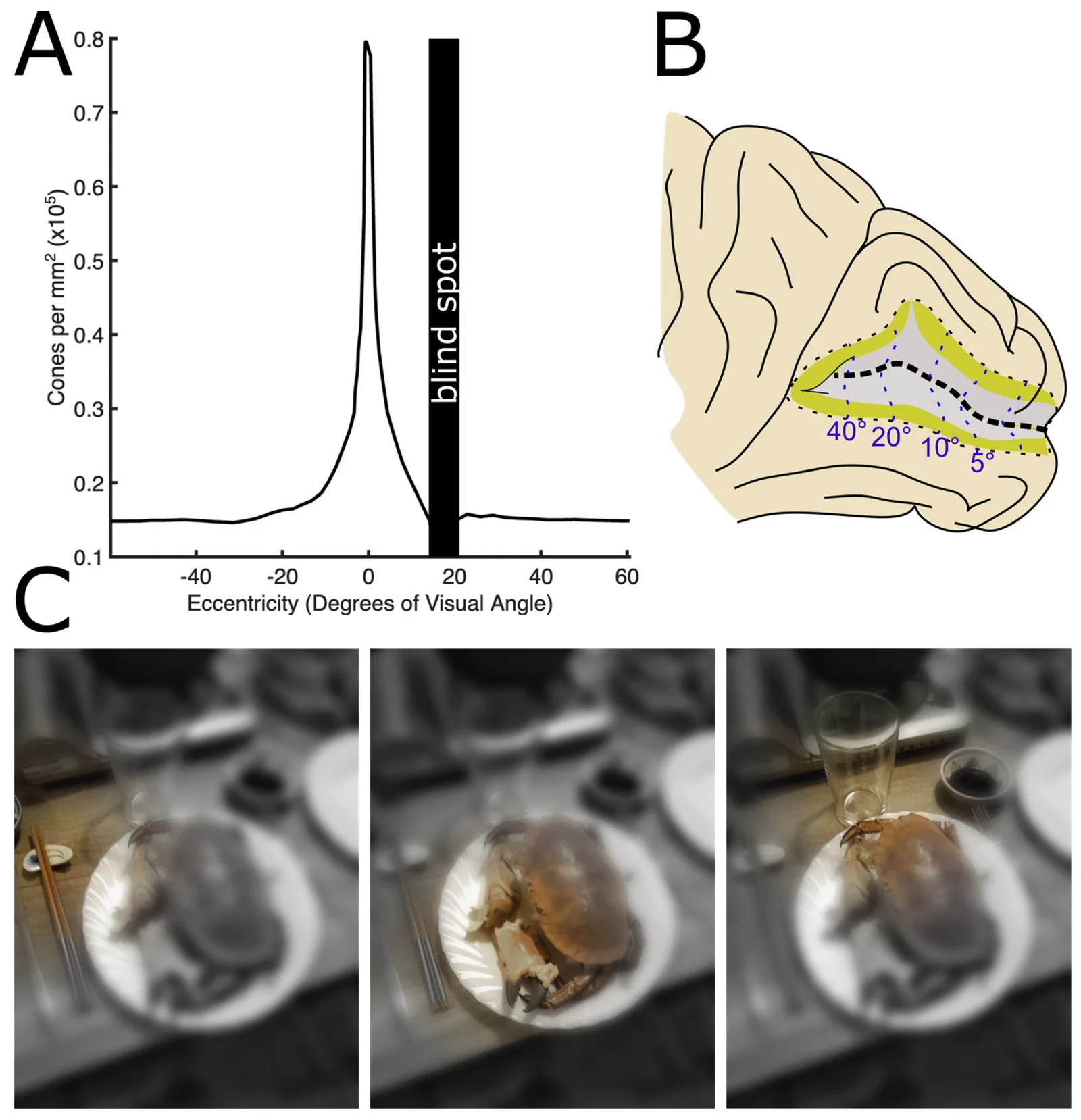
Limits of peripheral vision. (**A**) Distribution of photoreceptor density across retinal eccentricity, adapted from Østerberg (1935) [3]. The x-axis shows retinal eccentricity (degrees of visual angle), and the y-axis shows cone receptor density (cone/mm^2^). Cone density peaks sharply at the fovea and declines steeply with eccentricity. (**B**) Schematic representation of retinotopic organization in the primary visual cortex. Cortical regions receiving input from different retinal eccentricities are shown, illustrating the strong cortical magnification of central vision: a disproportionately large portion of the visual cortex is devoted to processing low eccentricities near fixation. Blue dashed lines and labels indicate retinal eccentricity (5°, 10°, 20°, and 40°), while the yellow-green region highlights the mapped cortical area. Adapted from Proj retine CGL cortex1 by Pancrat, Wikimedia Commons, licensed under CC BY-SA 3.0; modifications were made to the original. (**C**) Illustrative simulation of the perceptual consequences of reduced peripheral visual processing. The simulation is not quantitatively accurate and is included for conceptual purposes only. The three images show how the visual scene changes as fixation shifts across task-relevant regions while an observer is dining (from left to right, each panel corresponds to a different fixation location). Importantly, the pattern of degradation depends on fixation position, so peripheral information available at any moment differs across successive fixations. Photograph by one of the authors.

When we look at the world, we have the compelling impression of a rich and detailed visual scene extending far beyond the point of fixation. This phenomenology is striking because it appears fundamentally inconsistent with the known architecture of the visual system. High-acuity vision is limited to the fovea, a tiny retinal region subtending only a few degrees of visual angle [1]. This anatomical specialisation begins at the retinal level: cone photoreceptor density peaks dramatically in the fovea and declines steeply with retinal eccentricity (Figure 1A), dropping by more than an order of magnitude within the central few tens of degrees [2,3]. This peripheral undersampling is amplified by cortical organisation. Retinal inputs from central vision receive disproportionately greater cortical representation (Figure 1B), a phenomenon known as cortical magnification, whereby a large fraction of the primary visual cortex is devoted to processing the central visual field [4,5].

These anatomical constraints have profound perceptual consequences: as eccentricity increases, the precision and fidelity of visual processing progressively deteriorate. Spatial acuity decreases [6], contrast sensitivity changes [7], crowding increases [8,9], and chromatic discriminability declines [10,11,12]. Changes go beyond visual performance, as even the appearance of fundamental features such as brightness [13], hue and saturation [14,15,16], and spatial frequency becomes systematically distorted [17].

These limitations are well established in laboratory studies using simple stimuli. Yet in natural viewing conditions, observers rarely experience peripheral vision as severely degraded. While reading, text outside fixation does not appear dramatically blurred. When inspecting a textured wall, the periphery does not appear impoverished or statistically compressed. When looking at the sea, one does not perceive waves gradually disappearing with eccentricity. Instead, visual appearance seems strikingly uniform. Figure 1C illustrates how appearance should change across three saccades, based on our experimental knowledge of peripheral vision. However, we do not notice these changes, and we have the impression of detailed and rich peripheral vision.

This discrepancy constitutes a major theoretical problem. How can a perceptually rich visual experience arise from highly non-uniform sensory encoding?

An intuitive solution would be to assume that eye movements compensate for poor peripheral resolution by sequentially sampling the entire visual scene with the fovea [18]. However, decades of eye-movement research argue strongly against this explanation. During natural behaviour, observers fixate only a small subset of scene locations, mostly those relevant to current goals. In many real-world tasks, only a handful of objects receive direct fixation, while most of the environment remains exclusively peripheral [19,20,21] (for a review, see Hayhoe, 2017 [22]).

**Figure 2:**
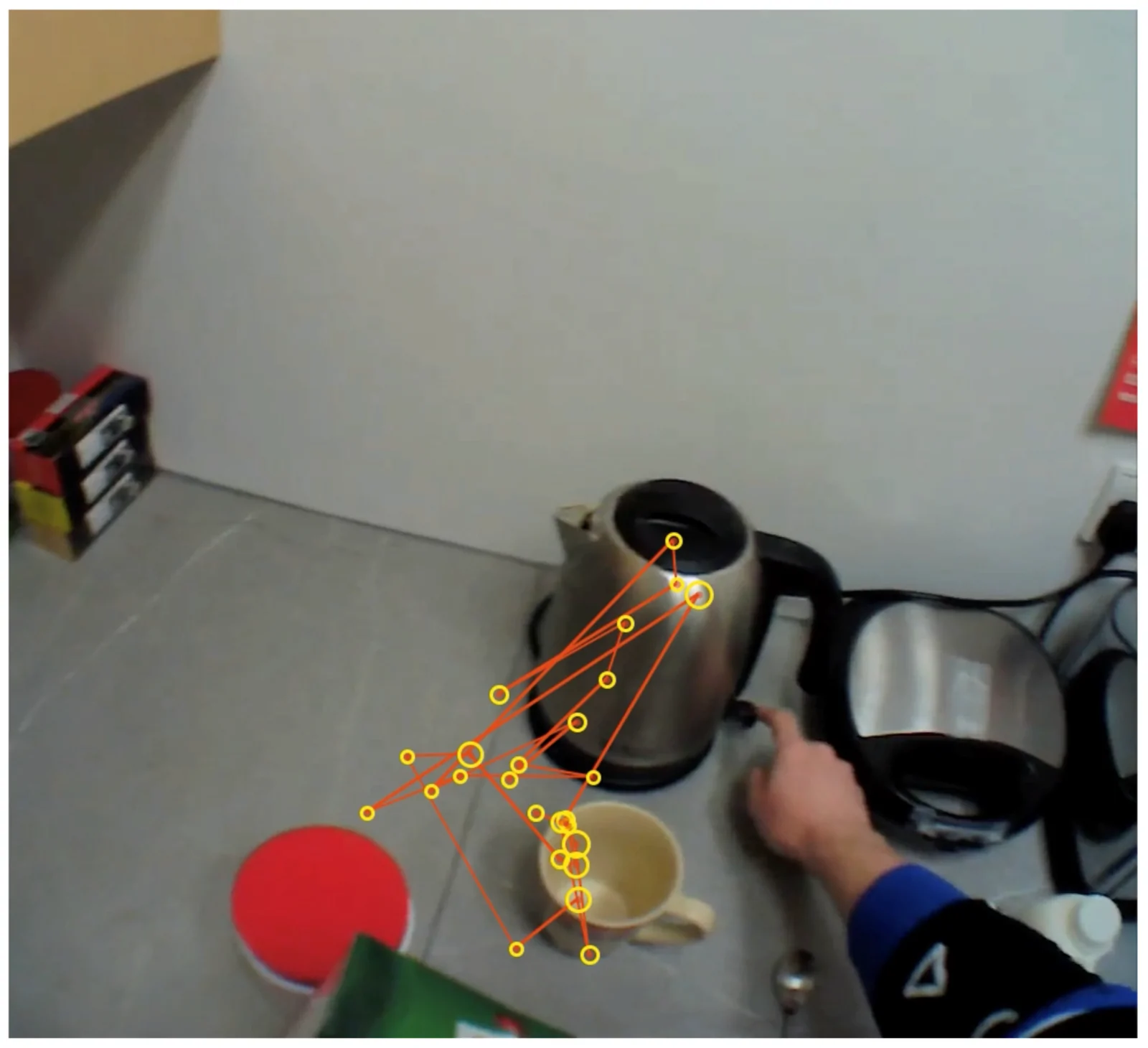
Fixations during preparation of a cup of tea. Example video frame with recorded eye movements superimposed. Yellow circles indicate fixation locations, with circle size scaled according to fixation duration; red lines connect successive fixations. Fixations accumulated over a 15-s interval are displayed to illustrate the distribution of gaze over time. Fixations are concentrated predominantly on task-relevant objects and locations, illustrating the selective allocation of gaze during natural behaviour. The video and eye-tracking data are original pilot data collected by the authors for separate ongoing work and are used here for illustrative purposes.

Recent advances in mobile eye-tracking technology have made it possible to record gaze behaviour during everyday activities outside the laboratory, providing a clearer picture of how vision operates in natural settings [19]. These studies reveal that fixation patterns are remarkably sparse and strongly centred on objects relevant to the ongoing task. For example, Figure 2 shows eye movements during the preparation of a peanut butter sandwich. Nearly all fixations fall on task-relevant objects, such as the jar, knife, bread, or hands, whereas large portions of the surrounding environment are never directly inspected and therefore remain only peripherally viewed.

This observation creates a striking paradox. Even though the visual system samples only a small fraction of the scene with high acuity, observers do not experience most of the environment as blurry, incomplete, or impoverished. Instead, peripheral vision appears phenomenologically rich and spatially uniform. Importantly, constructing a complete foveal representation of the environment through sequential fixations would require far more time and far more eye movements than are typically observed during natural behaviour. Yet the subjective impression of a detailed and coherent visual world arises continuously and almost immediately, long before enough fixations could have occurred to support such a reconstruction. This suggests that the apparent richness of visual experience cannot be explained simply by the accumulation of foveal snapshots across saccades.

In fact, it has been proposed that fixation eye movements are not primarily a mechanism for building a complete scene representation but for selecting and encoding task-relevant information into short-lived memory structures that support the ongoing action [23]. Within this framework, gaze shifts are tightly linked to “just-in-time” acquisition: information is acquired at fixation, used immediately for the current subtask, and discarded or overwritten unless it is needed again for action planning or control [19,24]. The world itself is used as an “outside memory” [25]. This implies that what is retained across saccades is not a detailed visual buffer of the scene but a minimal set of task-relevant variables anchored to recently fixated objects or scene locations.

**Figure 3:**
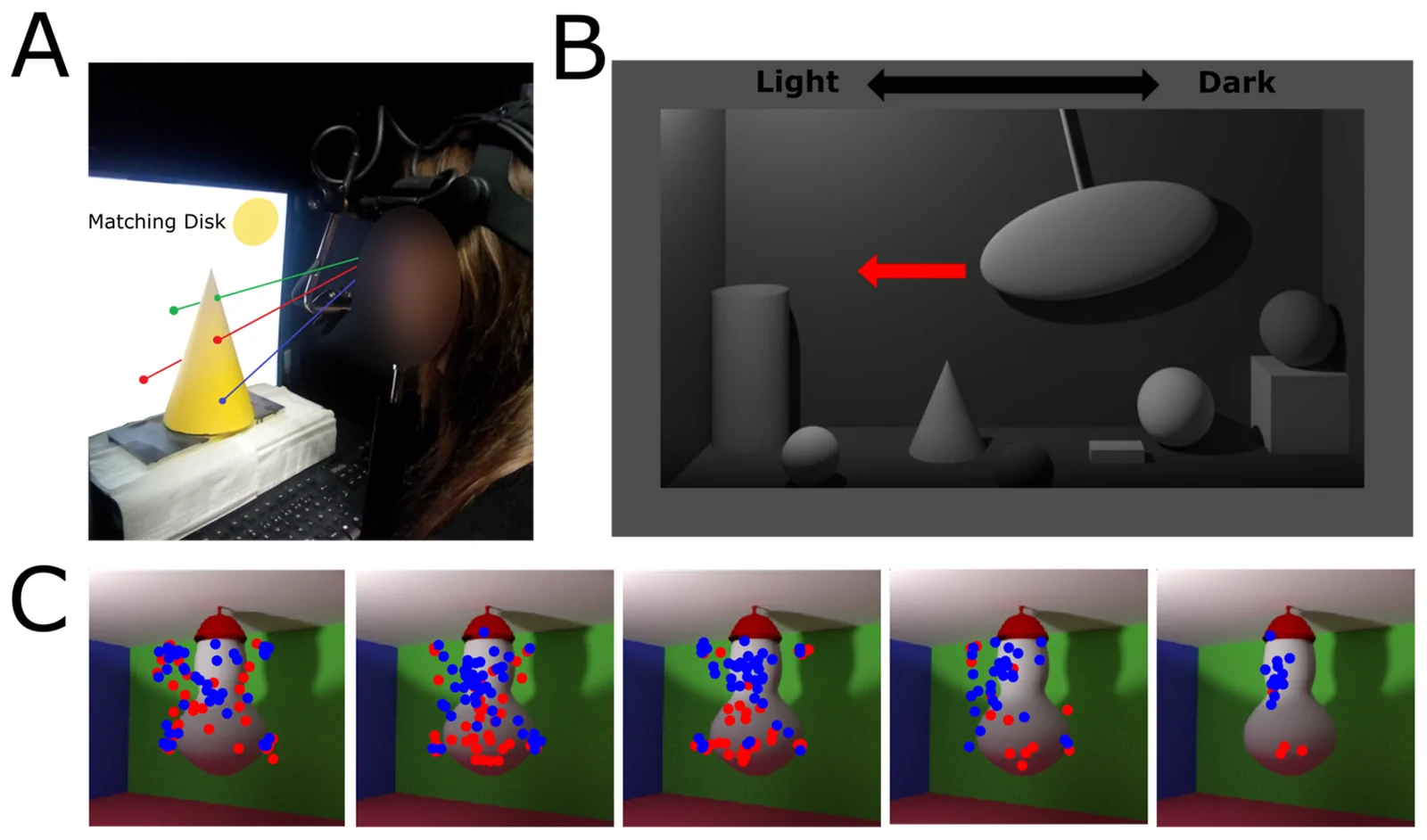
Fixation sampling within object surfaces. (**A**) Recording of fixations on real objects while participants were engaged in a lightness matching task. The setup was calibrated so that fixation positions in pixel units could be mapped back onto regions of the real objects. Participants were asked to adjust the matching disc presented in the top-right corner of the computer screen so that it appeared as if it were made of the same material as the natural object. The photo was taken from a similar experimental setup to that in Toscani and colleagues (2013) [26,27]. (**B**) Pendulum-like stimulus. The pendulum was rendered inside a virtual box with a left-to-right illumination gradient. It could move either from left to right or from right to left, following typical pendulum dynamics. The stimulus is the same as in Toscani et al. (2016) [33]. (**C**) Fixation changes with either softness or lightness judgments. The stimulus is a soft object hanging from the ceiling and poked from the back to induce a wobbling motion. Motion is stronger at the bottom, away from the point where the object is attached to the ceiling, providing more information about object softness. Illumination comes from above, providing a top-down gradient of information for estimating object reflectance. In this way, reflectance-diagnostic and softness-diagnostic information are effectively dissociated. Dots represent fixations on the object when participants were asked to judge object softness (red) or object lightness (blue). The images from left to right represent successive fixations (first, second, etc.). Initial fixations do not clearly differ across tasks; later fixations shift toward the lower portion of the object during softness judgments (where motion information is more informative) and toward the upper portion during lightness judgments (where more illumination-based information is available). The stimuli are the same as in Metzger et al., (2024) [28], but the fixations are from a different, individual, participant, from a pilot study.

This tight coupling between gaze and task demands extends even within individual object surfaces. When judging objects’ lightness (Figure 3A), observers preferentially sampled regions that were diagnostic for the required estimate, and their perceptual reports depended on which part of the object was fixated [26,27]. Further evidence comes from a study using a simple dynamic scene in which an elastic object fixed to the ceiling was struck (Figure 3C), and observers were asked to judge either stiffness or lightness [28]. Despite the task difference, initial fixations consistently landed near the centre of the object, suggesting an early, largely automatic component of gaze deployment that is relatively insensitive to task demands. After this initial phase, fixation behaviour shifted: later fixations increasingly targeted regions that were diagnostic for the current task, i.e., brightest regions for lightness [29,30] vs. deformation cues for stiffness [31]. This transition was accompanied by a corresponding effect of saccadic latency, with short-latency saccades producing task-independent landing positions, whereas longer-latency saccades reflected task-specific selection. Critically, this later, task-dependent phase of gaze control was the one most strongly associated with high perceptual performance, indicating that accurate judgments depended on late, task-aligned sampling rather than early exploratory fixations.

In a study investigating the dynamic aspect of eye movement selection for lightness judgments [32], observers viewed a continuously moving pendulum-like stimulus while eye movements were recorded (Figure 3B). Crucially, the pendulum moved across a room with an illumination gradient and could stop in either dimmer or brighter regions. Once it stopped moving, it was no longer shown, and participants were asked to match its lightness to a grey reference sample. Fixations consistently landed on the most diagnostically informative regions of the display (e.g., the brightest parts of the surface) across the entire duration of the trial. However, perceptual judgments were not determined by this overall sampling distribution. Instead, performance was specifically predicted by the luminance of the most recently fixated regions. Overall, the findings are consistent with a “just-in-time” mechanism in which task-relevant information is continuously selected in the periphery and encoded only at the moment it becomes behaviourally relevant: before the lightness matching response.

Furthermore, a large body of evidence has shown that integration across saccades is generally poor. Jonides, Irwin, and Yantis [34] used a paradigm in which participants viewed two successive dot arrays, each containing 12 dots. The key manipulation was that the second array was positioned so that, if information were combined across a saccade, the two displays would effectively align in external space and form a single larger configuration (a 25-location grid with one missing dot). The participants’ task was to report the location of the missing dot in this implied combined array. In the true saccade condition, the second array appeared during an actual eye movement, so that the first and second arrays fell on different retinal coordinates but the same spatial location. In the simulated saccade condition, no eye movement occurred; instead, the display change was mimicked while fixation was maintained. Crucially, if the visual system were integrating the two retinal snapshots into a unified representation across the saccade, observers should have explicitly experienced or reconstructed a complete 25-dot array and therefore show consistent and accurate detection of the missing location based on true perceptual fusion. What the results actually show, when reinterpreted in light of later work [35,36], is that such integration does not occur. Performance advantages in the true saccade condition do not reflect the construction of a fused, detailed array across eye movements. Instead, the two displays are not combined into a single coherent percept; observers do not maintain a veridical, item-by-item representation across the saccade. Rather, trans-saccadic memory behaves much more like visual short-term memory: low capacity (roughly 3–4 objects), abstract, and object-based rather than space-based [37]. Information survives the saccade, but only in compressed form.

Subsequent studies using more complex stimuli, such as plaid patterns, oriented gratings, moving dot displays, and coloured discs, have provided robust evidence for trans-saccadic integration [38,39]. Importantly, the resulting percepts are often consistent with statistically optimal cue combination: signals are weighted according to their relative reliability, with more reliable inputs contributing more to the final estimate, i.e., closer-to-optimal weighting of pre- and post-saccadic information [40]. However, similar to classic studies, integration seems to rely on a quickly decaying and maskable visual memory trace [39]. Findings of optimal cue integration across saccades are not necessarily inconsistent with the limited capacity of visual memory across saccades. Limited trans-saccadic memory does not imply that no information is retained or integrated across fixations, but rather that such integration may be selective. Information about task-relevant features or saccade targets can be retained and combined with post-saccadic input, potentially in a statistically efficient manner, without requiring a detailed representation of the entire visual scene to be maintained across eye movements.

This interpretation was reinforced by the change-blindness literature. Participants viewed natural scenes while making eye movements and were asked to memorise the details of the images and to detect object changes [24,41,42]. During a saccade—when vision is strongly suppressed [43]—an object in the scene was changed (for example, deleted it or rotated). If the visual system stored a rich representation from one fixation and integrated it with the next, such changes should have been immediately obvious. Instead, observers frequently failed to notice surprisingly large changes, even when they were instructed to memorise the scene and monitor for changes. Detection depended strongly on whether the changed object had been the saccade target or lay close to fixation [42,44].

More recent work provides further evidence that saccade targets receive privileged processing even before they are fixated. Kroell & Rolfs (2022) [45] showed that, during saccade preparation, defining features of a peripheral saccade target can influence visual processing at the current foveal location. Specifically, sensitivity to foveal orientation information was enhanced when it matched the orientation of the upcoming peripheral saccade target, suggesting that foveal processing anticipates features of the target before the eyes reach it. Subsequent work showed that the magnitude and temporal dynamics of this effect depend on the strength of the peripheral target signal [46]. Recent neuroimaging evidence further indicates that information about peripheral saccade targets can be decoded from foveal retinotopic cortex before fixation, consistent with feedback of target information to foveal visual representations [47]. Together, these findings suggest that perceptual continuity across eye movements may involve predictive interactions between peripheral and foveal representations, rather than relying exclusively on the integration of successive foveal snapshots (for a recent review, see Kroell & Rolfs, 2026 [48]).

Related object-based accounts propose that visual stability may depend on maintaining correspondence between selected objects across changes in retinal input, rather than on preserving a detailed representation of the entire scene. Object-file theories, for example, propose that object representations are indexed and updated as objects persist across changes in viewpoint or fixation [49,50]. Such accounts are compatible with the possibility that perceptual continuity is maintained primarily for selected objects rather than uniformly across the visual field.

Altogether, the reviewed evidence strongly suggests that rich visual experience cannot be explained simply by exhaustive foveal sampling. Several alternative explanations have been proposed (Table 1). One possibility is that we do not actually perceive rich detail in the periphery but merely believe—and report—that we do; in this view, reports of rich peripheral perceptual experience reflect a cognitive bias rather than a genuinely perceptual phenomenon. According to this account, observers overestimate what they perceive, resulting in a metacognitive bias, or “inflation,” of subjective confidence [51]. A second possibility is that peripheral visual appearance, at least at attended locations, is actively reconstructed through perceptual mechanisms that compensate for missing detail and correct peripheral distortions. We consider several potentially complementary mechanisms underlying such reconstruction. First, information sampled foveally may be extrapolated into peripheral representations, effectively extending high-acuity information beyond the point of fixation. Second, missing information may be inferred from prior knowledge and learned environmental regularities, consistent with predictive coding accounts [52,53] in which perception reflects the integration of sensory input with top-down predictions. Third, peripheral information may be represented in a compressed, ensemble-based format rather than as a collection of individually specified elements. For example, the Texture Tiling Model proposes that peripheral vision represents local regions through summary statistics over pooling regions that increase with eccentricity [54], consistent with the broader metamer framework showing that images with markedly different local structure can nonetheless be perceptually indistinguishable when they preserve relevant higher-order statistics [55]. Importantly, these possibilities need not be mutually exclusive: summary-statistic representations may provide the format in which at least some type of peripheral information is represented, while foveal extrapolation, prior knowledge, and object- and scene-level constraints may contribute to determining the content of those representations.

## 2. Is Rich Peripheral Experience Only Metacognitive Inflation?

**Figure 4:**
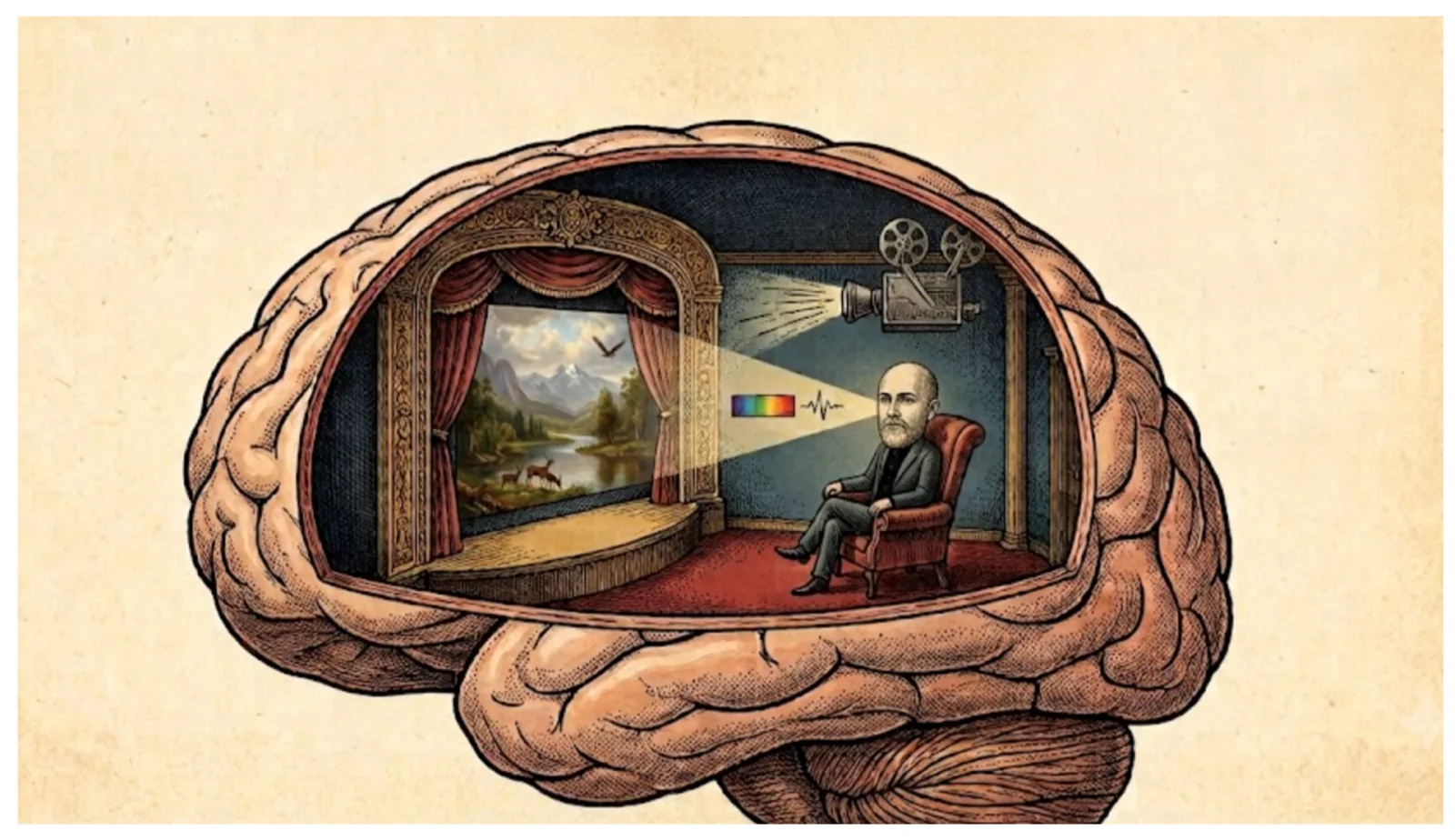
An illustration of the Cartesian Theatre. This philosophical metaphor depicts conscious experience as an internal stage or screen where sensory information is continuously projected. A central “inner observer” or homunculus sits within the brain, passively watching the stream of perception and reality unfold exactly as one would watch a film in a theatre. Created with the assistance of a generative AI model (Google Gemini) and subsequently edited by the authors.

The debate about the apparent richness of visual experience extends beyond perceptual science into the Philosophy of Mind, where it has been framed as a fundamental question about the nature of consciousness itself: does subjective experience genuinely contain the rich detail it seems to, or does introspection systematically mislead us? A highly influential position comes from Daniel Dennett, who argued that our sense of a richly detailed visual world is partly sustained by a mistaken theory of consciousness. Dennett challenged what he called the Cartesian Theatre [60] -the intuitive idea that perception involves a central inner observer inspecting a complete internal image of the world (Figure 4). According to this view, we implicitly assume that everything within our visual field, including the periphery, must be fully represented in consciousness at any given moment. Dennett argued that this intuition is deeply misleading. Rather than concluding that the visual system perceptually reconstructs all missing detail, Dennett proposed that we confuse the availability of information with its actual representation. Because information in the periphery can often be accessed later by shifting attention or moving the eyes, we infer that it was already present in experience. In his view, there is no mystery to solve regarding the rich impression of peripheral vision, because we simply do not have it; rather, we only believe we do, and this belief is what generates the apparent mystery.

This idea closely anticipates what contemporary vision science describes as *inflation*: the cognitive bias to overestimate the richness and precision of peripheral perceptual representations. Evidence comes from a study [51] where observers judged the orientation of a peripheral target Gabor patch that was either presented in isolation (uncrowded condition) or flanked by two additional gratings (crowded condition), the latter inducing classic crowding interference and therefore reducing access to target-specific information. The crowded condition was used a model of ecological peripheral vision. After each orientation discrimination response, participants provided confidence judgments, allowing the authors to quantify metacognitive sensitivity using meta-d′ [61]. The results showed that meta-d′ was significantly reduced in the crowded condition relative to the uncrowded baseline, indicating impaired metacognitive monitoring of performance when peripheral vision operates under crowding. Crucially, this reduction in metacognitive efficiency was accompanied by a systematic bias in subjective reports: in trials in which observers made incorrect orientation judgments, they nevertheless expressed higher confidence in the crowded condition compared to the uncrowded condition. In other words, crowding produced a metacognitive overconfidence bias, whereby observers overestimated the reliability of their perceptual decisions in precisely the conditions where sensory information was least reliable. These results were interpreted as a sign of inflation for peripheral vision.

The main problem with this study is that there is no direct comparison between central and peripheral vision, so it is not possible to conclude that there is overconfidence in metacognitive judgments in peripheral vision compared to central vision.

**Figure 5:**
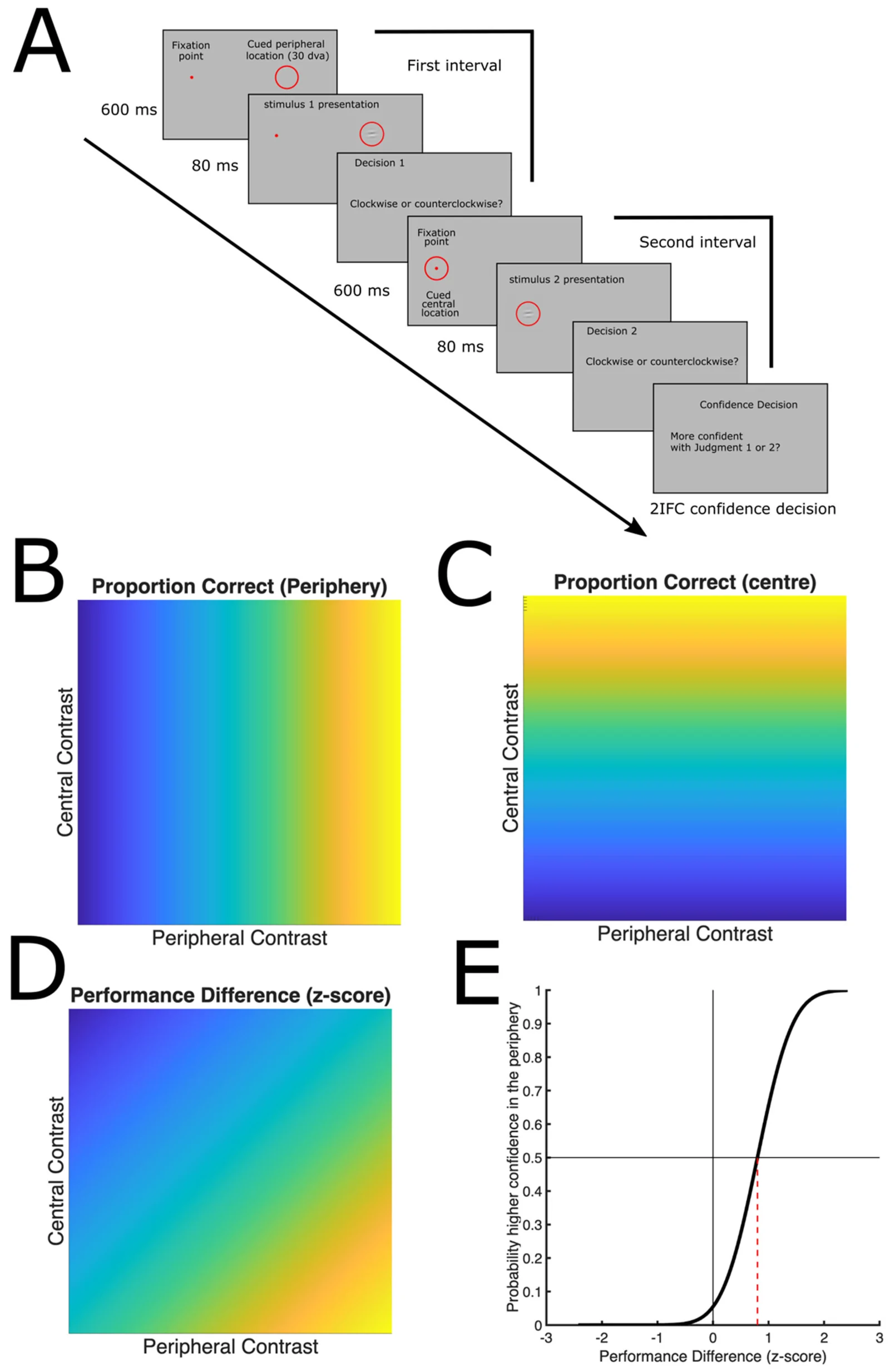
Underconfidence in peripheral vision. (**A**) Confidence forced-choice paradigm. Each grey rectangle illustrates an example of the computer display as presented to participants. The black text shown in the figure is included for explanatory purposes only and was not visible during the experiment. A trial consisted of two sequential perceptual intervals followed by a confidence judgment. In the first interval, a briefly presented Gabor patch appeared either at fixation (central vision) or at 30° eccentricity (peripheral vision), with the order randomised across trials. Participants judged whether the grating was tilted clockwise or counterclockwise. In the second interval, a Gabor patch was presented at the other location (central or peripheral), and participants again made an orientation discrimination judgment. After completing both perceptual judgments, participants performed a two-interval forced-choice (2IFC) confidence judgment, indicating which of the two orientation decisions they believed was more likely to be correct. The numbers shown to the left of the timeline (solid black line) indicate the presentation duration of each display. Displays without a specified duration remained on the screen until the participant responded. (**B**) Perceptual performance in peripheral vision. Heat map showing the proportion of correct orientation discrimination responses for the peripheral Gabor stimulus as a function of peripheral stimulus contrast (x-axis) and central stimulus contrast (y-axis). False colour indicates the proportion of correct responses. Performance increased systematically with increasing peripheral contrast and was independent of the contrast of the central stimulus. (**C**) Perceptual performance in central vision. Heat map showing the proportion of correct orientation discrimination responses for the central Gabor stimulus as a function of peripheral stimulus contrast (x-axis) and central stimulus contrast (y-axis). Axes and colour scale are identical to those in panel B. Performance increased systematically with increasing central contrast and was independent of the contrast of the peripheral stimulus. (**D**) Difference between peripheral performance and central performance. Axes are identical to those in panels (**B**,**C**). (**E**) Confidence judgments. Probability of reporting higher confidence in the peripheral judgment (y-axis) as a function of performance difference as shown in (**D**), on the x-axis. The dashed vertical line indicates the performance difference needed to yield equal probability of higher confidence for central and peripheral judgments. In case of no bias, it should correspond to zero performance differences. A value higher than zero indicates underconfidence in peripheral vision. Illustrations are adapted from Toscani et al. (2021) [62]. All data are simulated to provide a clean and immediate representation of the trends reported by Toscani et al. (2021) [62].

We designed a study in which confidence was directly compared between central and peripheral vision. In this study, participants completed a two-interval confidence forced-choice (2IFC) task designed to directly compare metacognitive confidence for central and peripheral perception (Figure 5A). In each trial, participants first performed two successive orientation discrimination judgments: in one interval, a briefly flashed Gabor patch appeared at fixation (central vision), and in the other interval, a Gabor patch appeared in the periphery (30° eccentricity), with order randomised across trials. For each presentation, participants judged whether the grating was tilted clockwise or counterclockwise. After making both orientation judgments, they performed a confidence comparison task, indicating which of the two perceptual decisions they believed was more likely to be correct, the central or the peripheral one. This design allowed the authors to compare confidence directly between central and peripheral judgments while controlling perceptual performance by adaptively adjusting stimulus contrast. Even when central and peripheral orientation discrimination performance was matched, observers were more likely to assign higher confidence to the central judgment, indicating peripheral underconfidence (Figure 5B–E). Thus, peripheral stimuli required substantially stronger sensory evidence before confidence in peripheral performance matched confidence in central performance. This result is difficult to reconcile with a general inflation account, which would predict peripheral overconfidence in this task.

More recently, the same research group has reported new preliminary findings showing peripheral underconfidence [63], effectively challenging the inflation theory. In work presented at a recent conference (Vision Science Society, 2025), they replicated the finding that observers adopt a more liberal detection criterion in unattended peripheral vision, even when provided with trial-by-trial feedback or explicitly informed about this bias. However, when perceptual performance was matched across eccentricities, confidence decreased with increasing eccentricity, indicating underconfidence, rather than overconfidence, for peripheral perceptual decisions.

However, the picture appears to be more complex than simply underconfidence in peripheral vision. Metacognitive biases seem to depend on the type of task and whether it capitalises on the strengths or weaknesses of peripheral processing. While studies using fine orientation discrimination have reported underconfidence when perceptual performance is matched across eccentricities, a different pattern emerges for tasks in which peripheral vision has a natural advantage [64]. We asked whether overconfidence would re-emerge in a task that relies on global scene processing, a function for which peripheral vision is particularly well suited. Participants viewed natural scenes through either a central window or a peripheral-only display (central scotoma) and categorised each scene as a desert, beach, mountain, or forest. They then completed a two-interval confidence forced-choice judgment, indicating in which interval they were more confident. Task difficulty was manipulated by varying the size of the window and scotoma, thereby matching performance across central and peripheral conditions. Despite this performance matching, participants consistently exhibited greater confidence in their peripheral judgments.

These findings suggest that metacognitive biases are not fixed properties of peripheral vision but instead depend on the match between the task and the functional specialisations of the visual field. Peripheral vision is relatively poor at discriminating fine spatial details but excels at extracting coarse, global information and rapidly detecting objects and scene gist. Confidence judgments appear to reflect these ecological strengths and limitations. Thus, rather than exhibiting a general tendency toward either overconfidence or underconfidence in the periphery, observers seem to calibrate their confidence according to the type of information that peripheral vision is optimised to process.

However, taken together, the results demonstrate that a cognitive bias account such as “inflation” cannot fully explain the subjective impression that peripheral vision appears rich and detailed, particularly given that performance on fine-detail judgments in peripheral vision is markedly impaired. What, then, underlies this apparent richness?

We propose that at least two complementary mechanisms contribute to the experience. First, there is the use of information from central vision to extrapolate or “fill in” peripheral content, whereby foveal input and recent high-resolution samples are integrated and extended across space to generate a more complete percept. Second, perceptual experience is shaped by strong prior knowledge about the structure of the visual world: the visual system actively completes missing information based on learned regularities and scene-level expectations, consistent with predictive coding accounts of perception. Together, these mechanisms suggest that the richness of peripheral experience is not only a metacognitive illusion or reporting bias, but at least in part arises from constructive processes that combine limited sensory input with contextual extrapolation and prior-driven completion.

## 3. “Filling Out” Peripheral Vision: Centre-to-Periphery Extrapolation

Strong evidence for “filling-out” processes comes from the uniformity illusion, which shows that fine peripheral details can be perceptually overwritten by the central structure. (We use the term filling-out to refer specifically to the extrapolation of information from foveal to peripheral vision. This terminology distinguishes the direction of the proposed information transfer from classical filling-in, which generally refers to the perceptual completion of regions in which sensory information is absent or degraded. However, we do not intend this distinction to imply separate underlying mechanisms: foveal-to-peripheral filling-out may represent a particular form of, or rely on mechanisms shared with, perceptual filling-in).

The uniformity illusion [56] is a phenomenon where peripheral stimuli appear to take on the identity of stimuli presented in central vision. In the original study, Otten and colleagues (2017) [56] measured this effect by presenting participants with displays in which central and peripheral regions contained systematically different visual features (e.g., orientation, shape, luminance, motion, texture, or identity). Participants were instructed to maintain fixation on the centre of the display and view the stimulus for an extended period. In each trial, the central and peripheral elements differed at onset, but over time participants were asked to indicate when the display appeared completely uniform, meaning that the peripheral stimuli were no longer distinguishable from the central stimuli. Across experiments, the authors manipulated the feature differences between the centre and the periphery and quantified the proportion of trials in which participants reported the percept of uniformity, as well as the time required for the uniform percept to emerge. They found that, across multiple feature domains, observers often reported that peripheral stimuli had changed to match the central stimuli, even though no physical change occurred in the display.

The uniformity illusion establishes a change in reported peripheral appearance but does not by itself identify the computations producing this change or their neural implementation. Follow-up studies have therefore asked whether the perceptual effect is accompanied by changes in early sensory representations or instead depends on processing at later stages. Follow-up studies have investigated the level of processing at which this effect arises. Suárez-Pinilla et al., (2018) [65] aimed to outline the neural mechanisms behind the uniformity illusion using after-effects as an index of perceptual processing. Their results suggest that perceptual filling-out associated with the uniformity illusion occurs at later stages of visual processing, beyond V1 and V2, where more integrated perceptual representations are formed. They used an orientation adaptation paradigm designed to induce the illusion with arrays of Gabor patches. After adaptation, the stimulus array was removed, and participants were asked to report the orientation (tilt) of a single peripheral Gabor. The tilt aftereffect followed the physically presented adapting orientation, rather than the globally perceived orientation reported under the uniformity illusion, even when the illusion had been experienced for extended periods. These results suggest that the uniformity illusion does not reflect changes in early sensory encoding in V1, given that tilt aftereffects are widely taken as an index of adaptation in early orientation-selective mechanisms [66,67], but is more consistent with higher-level processing that does not directly alter early visual adaptation. However, the level of the visual system at which the uniformity illusion is implemented appears to depend on the type of perceptual feature that is being “filled out.” If we assume that the uniformity illusion relies on mechanisms akin to classical perceptual filling-in [68], then a useful parallel can be drawn with a well-established distinction in the neurophysiology of filling-in phenomena. In general, filling-in refers to the brain’s tendency to generate a percept of uniformity in regions where the sensory signal is degraded or absent, effectively “completing” missing information based on surrounding context. Critically, evidence from neurophysiology and neuroimaging suggests that this completion does not always occur at a single, fixed stage of visual processing. For low-level features such as colour or luminance, neural correlates of perceptual filling-in have been observed as early as V1 [69]. In contrast, for more complex properties such as texture, related filling-in effects appear to emerge only at later stages, including extrastriate areas such as V2 and beyond [68,70]. In fact, Otten et al. (2026) [71] were able to show after-effects of the uniformity illusion using a grid in which a peripheral patch changed in colour and size. Participants fixated the centre of a display in which the central and peripheral regions differed in colour or element size. After several seconds, many observers experienced the uniformity illusion. Once participants reported this illusion, the central region was changed to match the original peripheral pattern, making the entire display physically uniform. If the illusion disappeared immediately, observers should have perceived this new display as uniform. Instead, many continued to perceive the periphery according to the previous illusory appearance, and this persistent illusion also biased their subsequent reproductions of the peripheral colour or size and slowed their responses. These findings indicate that the uniformity illusion is not simply a failure to attend to the periphery but reflects an active perceptual process that can persist for several seconds after the inducing stimulus has been removed.

Regardless of the precise stage at which the uniformity illusion is implemented, it is clear that central and peripheral representations do not operate independently. A number of studies have shown that information from peripheral stimuli can be represented in the foveal cortex and can influence subsequent perception. One of the first pieces of evidence came from Williams and colleagues (2008) [72], who used functional MRI during an object recognition task and found that object category could be decoded from BOLD activity in the foveal retinotopic cortex even when the objects themselves were presented in the visual periphery. This finding suggested that peripheral information is fed back to cortical regions normally representing central vision. The behavioural relevance of these foveal representations was later demonstrated by Yu and Shim (2016) [73]. Participants discriminated a peripheral target (either a three-dimensional shape or an oriented grating) while a briefly presented, masked “foil” appeared at fixation. Peripheral discrimination improved when the foveal foil matched the peripheral target, indicating that foveal representations can play a functional role in peripheral visual perception. A similar result has been found for faces: the presence of a centrally presented face that was similar to a peripheral face improved discrimination of the peripheral face’s identity [74]. This again suggests that foveal representations can facilitate the processing of peripheral stimuli, possibly by providing expectations or perceptual templates that guide the interpretation of ambiguous or degraded visual information in the periphery. More recently, Kroell and Rolfs (2022) [45] proposed that the foveal retinotopic cortex receives information from the peripheral cortex through predictive remapping, suggesting that central representations may anticipate the consequences of future saccades even in the absence of eye movements. Together, these findings suggest that interactions between central and peripheral vision can improve perceptual accuracy rather than simply bias perceptual appearance.

Evidence that these interactions are mediated by early visual cortex comes from a transcranial magnetic stimulation study [75]. Applying TMS over the occipital cortex to interfere with foveal processing impaired discrimination of stimuli presented in the visual periphery, providing causal evidence that cortical regions representing central vision contribute to peripheral perception. Such findings are particularly relevant to the uniformity illusion, as they demonstrate that information represented at the fovea can influence the processing of peripheral stimuli through feedback or recurrent interactions.

**Figure 6:**
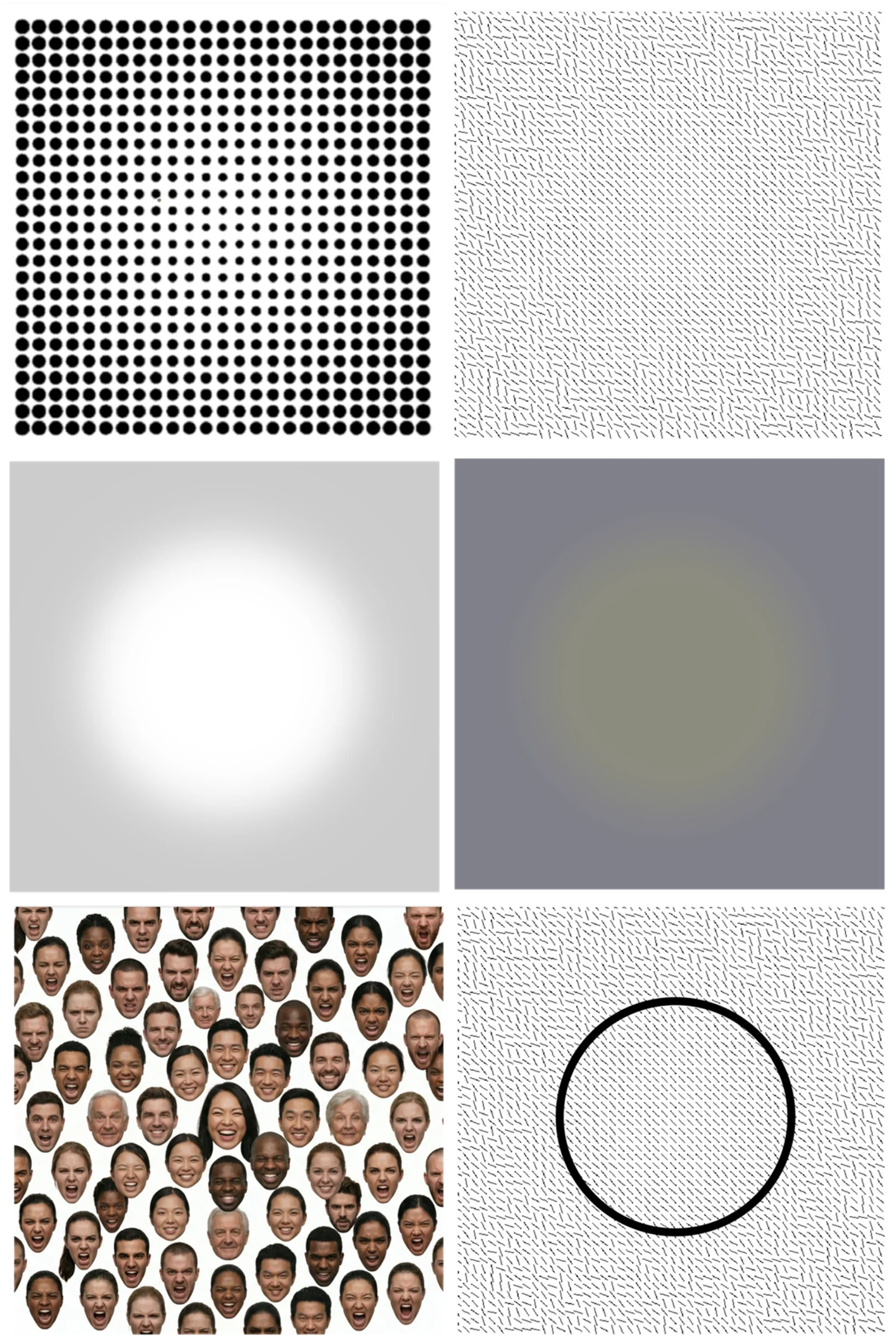
Examples of the uniformity illusion. The figure illustrates how perceptual information in central vision can bias or “regularise” the appearance of peripheral content across a range of stimulus types. For simple textures, such as dot patterns or oriented line fields, properties defined at fixation (e.g., dot size or orientation) tend to be extrapolated into the periphery, leading to a perceptual impression in which peripheral elements appear more similar to the central ones (e.g., smaller dots or more uniformly oriented lines). The same type of influence extends to basic perceptual dimensions, including luminance and colour, where central values can dominate or smooth perceptual appearance across the visual field. Crucially, this filling-out process is not limited to low-level features: it also affects more complex, high-level properties such as facial expressions in crowd scenes, where the perceived emotion of peripheral faces can be biased toward the dominant expression in the central region. However, this effect appears to be constrained by segmentation and perceptual boundaries. In particular, the presence of a strong contour or edge can prevent the spread of central information into the surrounding region, as illustrated in the bottom-right panel, where the circular boundary preserves local peripheral structure. The simple texture examples are inspired by work by Otten and colleagues (2017) [56], while the crowd-face stimuli are inspired by a study by Zoghlami and Toscani (2023) [57]. These versions of the illusion differ from those originally presented by Otten and colleagues (2017) [56] in that the transition between central and peripheral features is smooth. We chose this because we found that the illusion emerges faster.

While the uniformity illusion has been demonstrated for a wide range of visual properties (Figure 6), interactions between central and peripheral vision are not always expressed as a homogenisation of appearance. One example is the honeycomb illusion [76], in which a physically uniform texture gives rise to a strikingly non-uniform percept. The stimulus consists of a regular honeycomb pattern in which each hexagonal cell contains small line segments (“barbs”) attached to its vertices. Although these barbs are physically present throughout the display, they are readily visible only around fixation and appear to disappear in the periphery, whereas the hexagonal lattice itself remains visible. As a result, observers perceive a central region that differs from the surrounding texture despite the display being perfectly uniform. Moreover, the illusion persists despite repeated eye movements, indicating that the visual system does not simply integrate high-resolution information acquired across successive fixations to create a uniform percept of the scene. Thus, a physically uniform stimulus can appear perceptually heterogeneous because of the differences in how central and peripheral vision encode fine spatial detail.

Furthermore, information sampled in central vision does not always facilitate perception in the periphery. In the central region interference with periphery (CRIP) effect [77], observers discriminate the orientation of line elements presented in the periphery while irrelevant line patterns are shown in the centre of the display. Rather than improving performance, the central pattern impairs discrimination of the peripheral target. The interference increases as the separation between the central and peripheral regions decreases and is strongest when the two regions contain similarly oriented elements. Surprisingly, performance is worse when the central and peripheral lines have the same orientation than when they differ. These findings demonstrating that foveal information can compete with, rather than support, peripheral processing.

These seemingly contradictory observations highlight the importance of studying central–peripheral interactions using complex natural stimuli. This is particularly important because the uniformity illusion raises a fundamental problem for natural vision. If information were propagated indiscriminately from the fovea into the periphery, the appearance of one object could erroneously influence the appearance of neighbouring objects with different colours, textures, or materials.

Coincidentally, in the same year that the uniformity illusion was first reported, we were investigating the influence of luminance at fixation on peripheral brightness perception using photorealistic three-dimensional rendered scenes [32]. Participants viewed objects composed of a single material (e.g., marble), whose luminance varied naturally because of shading. Using a gaze-contingent display, we forced observers to fixate either a relatively bright or a relatively dark region of the object while they matched the brightness of a peripheral region using an adjustable comparison disc. Importantly, participants were instructed to ignore the scene and judge only the peripheral target. Nevertheless, their matches were systematically biased by the luminance at fixation: the peripheral region was judged darker when fixation was constrained to a dark part of the object than when fixation was constrained to a bright part.

Crucially, this bias disappeared when observers fixated a location outside the object while retinal luminance at fixation and the eccentricity of the peripheral target were kept constant. Thus, the same foveal luminance no longer influenced peripheral appearance when it belonged to a different object. This finding resolves one of the principal objections to foveal extrapolation in natural scenes. Rather than spreading indiscriminately across the visual field, the influence of foveal information appears to be constrained by object boundaries. Interestingly, this conclusion is consistent with subsequent work showing that the original uniformity illusion is also disrupted by a clear border separating the central and peripheral regions (Figure 6, bottom right), further suggesting that perceptual homogenisation is constrained by object segmentation.

We therefore propose that, at least for perceived brightness, foveal luminance biases the appearance of peripheral regions only when there is good reason to infer that they belong to the same physical object—that is, when they are likely to share the same surface reflectance despite local variations in illumination. Such an object-based mechanism would compensate for the unequal spatial resolution of central and peripheral vision while preventing the inappropriate transfer of appearance between distinct objects.

**Figure 7:**
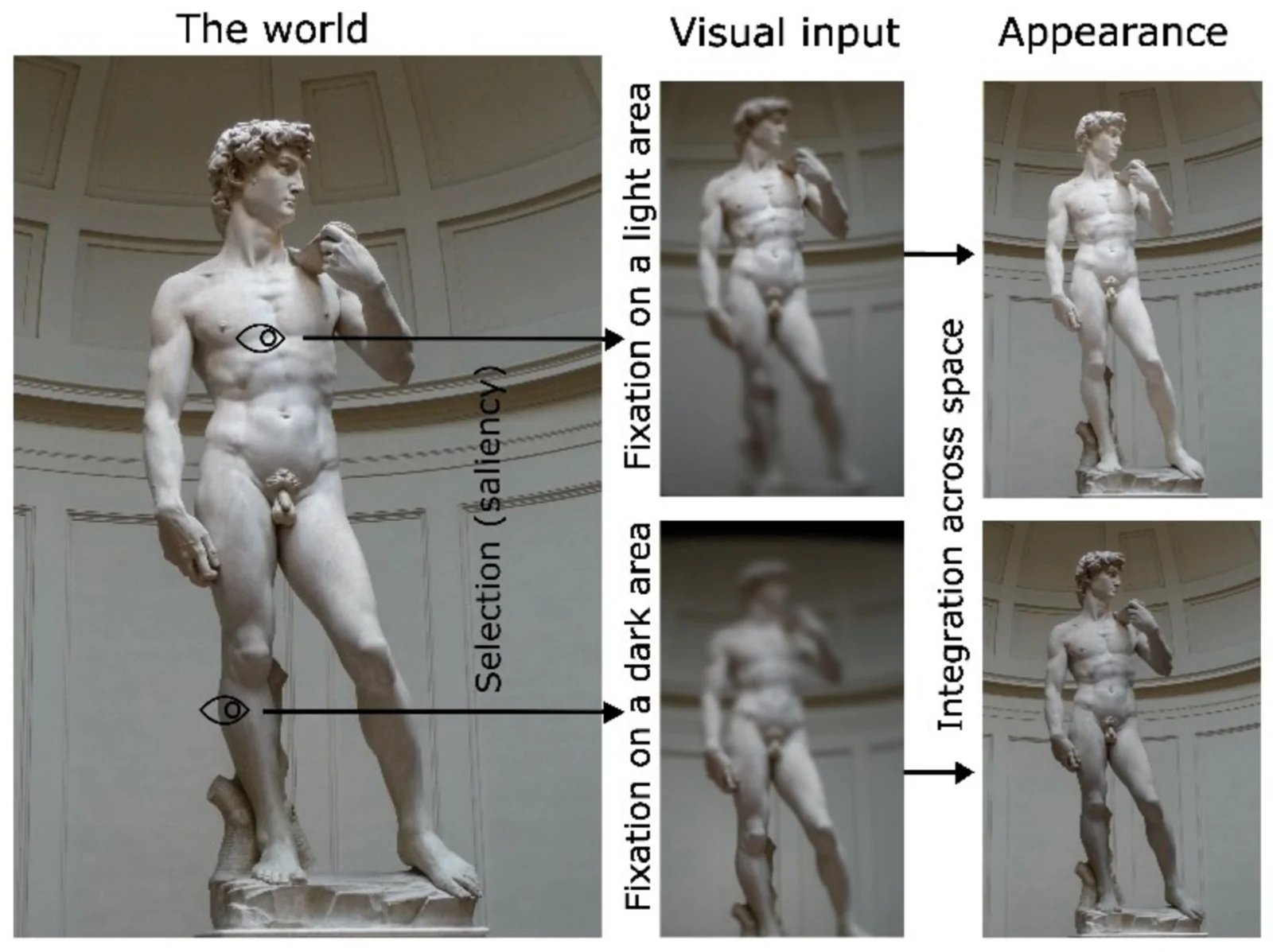
Active sampling and foveal-to-peripheral extrapolation support a coherent percept of object appearance. The **left** panel illustrates the physical world, where an observer can selectively sample different regions of an object by directing gaze. Because visual acuity is highest at fixation, the retinal input differs depending on the selected region. Fixating a bright region provides a high-resolution sample of the object’s luminance under that illumination, whereas fixating a darker region provides a different local sample. These foveal samples are then integrated with lower-resolution peripheral information to generate a stable percept of the object’s appearance. The (**upper**) pathway illustrates fixation on a bright, highly informative region: information sampled in the fovea biases the interpretation of the less detailed peripheral representation, resulting in a brighter overall appearance. The (**lower**) pathway illustrates fixation on a darker region: despite the different local input, the same object-based integration process leads to a darker perceived appearance. Thus, active gaze selection determines which regions provide the strongest constraints on object appearance, and foveal information can be extrapolated across the object to compensate for the limited resolution of peripheral vision. Importantly, this extrapolation is proposed to operate within object boundaries, allowing the visual system to enhance perceptual constancy while avoiding inappropriate transfer of appearance information between distinct objects. Image of Michelangelo’s David. Adapted from a photograph by Jörg Bittner Unna, via Wikimedia Commons, licensed under CC BY 3.0. Changes were made to the original.

This proposal also offers a functional account of active vision. When estimating the lightness of an object, observers preferentially direct their gaze towards its brightest regions, which provide the most informative samples of surface reflectance [26]. If the luminance sampled at fixation also biases the perceived brightness of other regions belonging to the same object, this strategy would promote perceptual uniformity across the object’s surface while simultaneously enhancing the perceived differences between objects with different reflectances (Figure 7).

Whether our findings generalise to visual features other than lightness remains an open question. However, there is evidence that centre-to-periphery interactions extend beyond simple surface properties and influence the perception of complex visual ensembles. For example, the emotional expression of faces in a crowd is not necessarily processed by analysing each individual face independently. Instead, the visual system can rapidly extract summary statistics from groups of faces, forming a synthetic impression of the overall emotional content of a crowd even when observers are unable to report the detailed features of individual faces (ensemble coding—Haberman & Whitney, 2012 [78]). We investigated whether such ensemble representations are influenced by interactions between foveal and peripheral vision [57]. Participants viewed arrays of faces and were required to adjust the emotion in a continuum from sad to happy to match the perceived emotion of a target face presented in the periphery. The results showed that the perceived emotion of the peripheral target was systematically biased by the emotion of the face viewed at fixation. Importantly, this effect was not restricted to conditions in which fixation was experimentally controlled. When participants were allowed to freely explore the displays, their spontaneous eye movements produced the same pattern of centre-to-periphery influence: the emotional information sampled at fixation biased overall perceived emotion of the crowd. As emotional faces attract more fixations than neutral faces [79,80,81], our results suggest that the overall emotion of a crowd of faces may be exaggerated. Indeed, we found that this selective fixation strategy appeared to influence the perceived overall emotion of the crowd: groups of sad faces were perceived as sadder than their average, whereas crowds of happy faces were perceived as happier. This is an example of how interactions and differences between central and peripheral vision can mediate ensemble coding.

Extrapolation within an object surface, a texture, or an ensemble is functionally reasonable because it amplifies the properties of selected regions and promotes perceptual uniformity. However, it needs to be constrained within relatively uniform entities; otherwise, as discussed previously, it would promote confusion between objects rather than helping us to distinguish and recognise them. This raises the question of how the richness of peripheral vision and perceptual uniformity are maintained when fixating one object while other, different objects remain in peripheral vision. This is a common situation, as we typically fixate task-relevant objects while leaving the surrounding environment in the periphery (Figure 2). We argue that, in these cases, at least when attending to a peripheral object, we fill in its properties based on prior knowledge. In the next paragraph, we discuss evidence showing how typical colours influence the perceived colour of peripheral objects and how the visual system can learn how the size of an object presented peripherally will appear when viewed directly.

## 4. Effects of Prior Knowledge on Peripheral Appearance

One influential idea in vision science is that sensory signals are interpreted based on prior knowledge about the world. This influence is particularly evident when sensory information is ambiguous and the visual system must infer the most likely interpretation of the scene. A classic example is the perception of shape from shading. Because natural illumination typically originates from above, observers develop a strong prior that brighter regions on a surface are more likely to reflect changes in illumination than changes in three-dimensional shape. Ramachandran (1988) [82] demonstrated that this assumption systematically influences perceived shape: identical shading patterns can be perceived as convex or concave depending on their orientation relative to the expected direction of illumination. Such findings illustrate that visual appearance reflects an interaction between sensory evidence and prior assumptions about the physical world. This idea has been formalised within Bayesian theories of perception, in which the visual system combines uncertain sensory signals with prior probabilities acquired through experience to generate the most probable interpretation of the environment [83,84]. According to this framework, priors are not a cognitive correction applied after perception but an integral component of perceptual inference, shaping the contents of visual experience itself.

A well-studied example of such a perceptual prior is the memory colour effect: the tendency for familiar objects to appear biased towards their characteristic colours. For instance, observers judging the colour of a banana as neutral typically shift its appearance towards bluish hues, revealing an influence of the remembered yellow colour associated with bananas [85]. Similar biases have been demonstrated for a variety of colour-diagnostic objects and have been shown to depend on learned associations and familiarity [86,87,88]. Importantly, memory colour is not simply an effect of recognition or verbal knowledge about objects; it alters the perceived appearance of the stimulus itself.

The influence of memory colour can be understood within a Bayesian framework, in which perceptual estimates arise from the combination of sensory evidence and prior expectations about the world [83,89]. In this framework, the relative contribution of each source depends on its reliability: when sensory information is precise, perception is dominated by the incoming signal, whereas when sensory information is noisy, prior expectations exert greater influence. Witzel, Olkkonen, and Gegenfurtner (2018) [90] formalised the memory colour effect in precisely this way, modelling object colour appearance as the weighted integration of a noisy sensory estimate with a prior centred on the object’s typical colour. A direct consequence of this account is that memory colour effects should become stronger when chromatic signals are less reliable, as occurs in peripheral vision.

Consistent with this Bayesian prediction, Metzger, Valsecchi, and Toscani (2026) [58] showed that memory colour biases are enhanced in peripheral vision. The study was motivated by the specific prediction that, because peripheral chromatic signals are less reliable, the visual system should increase its reliance on colour priors. Using colour-diagnostic objects presented either foveally or at 10° eccentricity, the authors measured the colour settings required for objects to appear grey. Peripheral grey settings were systematically shifted further away from the objects’ typical colours than central grey settings, indicating a stronger influence of memory colour in the periphery. Importantly, this enhancement was not simply an eccentricity-dependent bias: peripheral vision also showed greater variability in grey settings, providing a direct measure of increased perceptual uncertainty. These findings suggest that the functional role of memory colour extends beyond its previously proposed contribution to colour constancy. Although object-colour knowledge has been shown to provide a small but measurable benefit in maintaining colour stability across changes in illumination [91], prior knowledge may contribute substantially to the rich and colourful appearance of the peripheral visual field.

**Figure 8:**
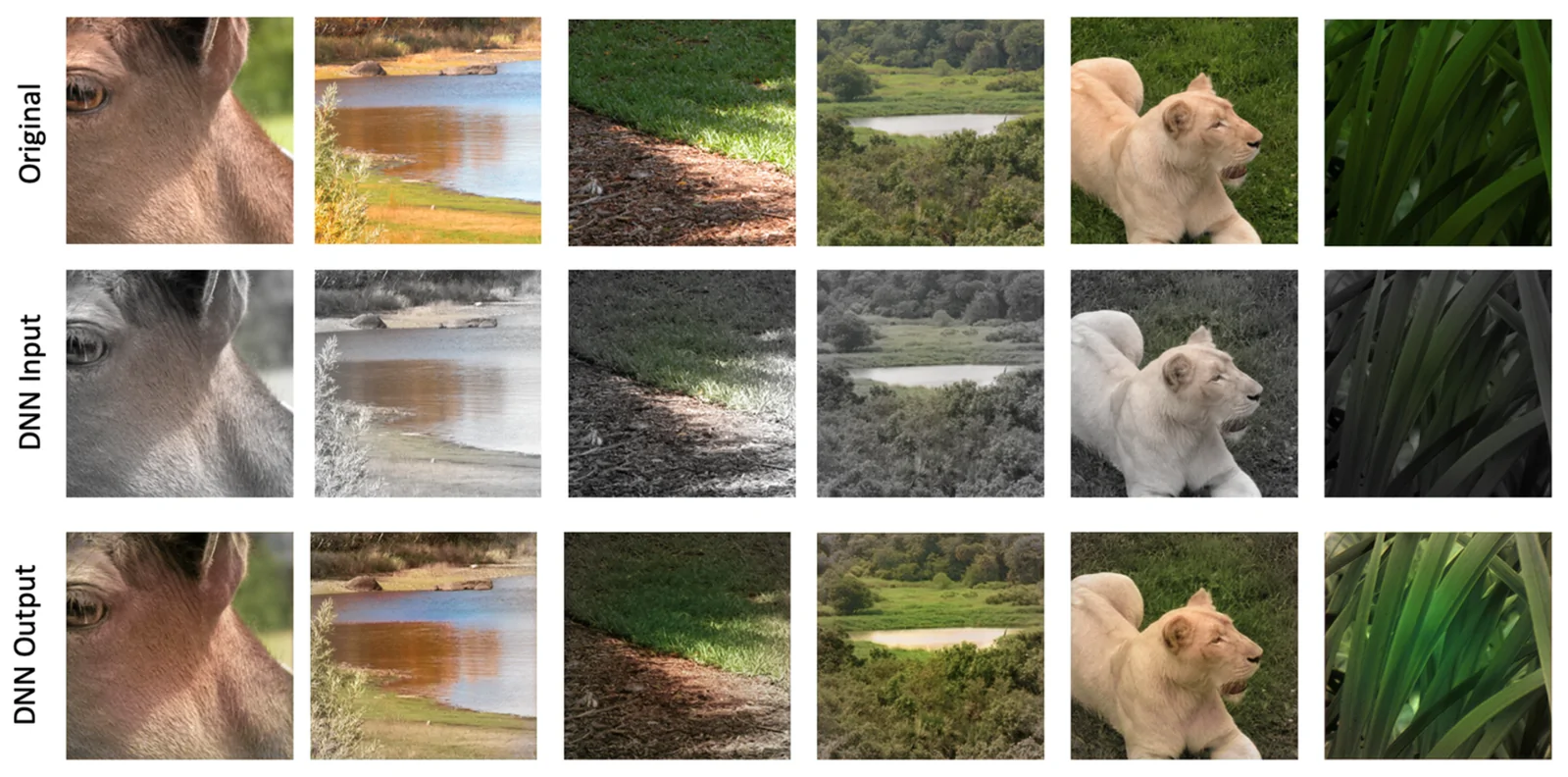
Computational reconstruction of peripheral colour information using a convolutional neural network. To simulate the loss of chromatic information in peripheral vision, colour images from the McGill colour image database were transformed into CIELAB space, and chroma (the a and b components) was progressively reduced as a function of eccentricity using a Gaussian-smoothed radial mask, while preserving luminance (L). The Gaussian decay was chosen such that chroma gradually decreased with eccentricity and reached zero in the far periphery, resulting in effectively achromatic images outside the central region—σ was set to ¼ of the image side size. The resulting foveated images were used as inputs to a U-Net DNN trained to reconstruct the original colour images. The network was trained on 256 × 256 pixel image patches extracted from 766 natural images, with 64 patches per image and a batch size of 16. The U-Net architecture consisted of a single encoder–decoder level (encoder depth = 1), with a regression output layer predicting continuous colour values. Training used stochastic gradient descent with momentum (0.9), an initial learning rate of 0.1 with stepwise reduction, L2 regularisation (10^−4^), and 100 epochs. The trained network successfully reconstructed plausible peripheral colour distributions from degraded input images. Reconstruction examples show the original images (top line), the simulated peripheral colour degradation (middle line—DNN input), and the colour reconstructed by the network (third line—DNN output). The simulation is not published but was presented at the European Conference on Visual Perception in the 2025 edition, within an oral contribution titled “Colour perception in peripheral vision is particularly affected by prior knowledge”.

Such a mechanism can operate only if object–colour associations in the natural environment are sufficiently stable and predictable. Otherwise, prior-based reconstruction would frequently generate perceptual errors, leading to noticeable mismatches between the appearance of objects in central and peripheral vision. Evidence that these statistical regularities are indeed strong enough comes from computer vision. Modern colourisation algorithms can reconstruct highly plausible colours from greyscale images by exploiting statistical associations between object identity, scene context, and colour, often producing results that are difficult to distinguish from natural photographs (e.g., [92,93,94]). Their success demonstrates that natural scenes contain sufficiently reliable object–colour regularities to support accurate inference of missing chromatic information. A remaining question concerns how such inferred peripheral colour information is integrated with the more reliable chromatic signals available at the point of fixation. To explore whether the statistics of natural scenes are sufficient to support such integration, we trained a Deep Neural Network (DNN) to reconstruct colour after selectively removing chromatic information from the peripheral regions of natural images while preserving foveal colour. The DNN learned to restore plausible peripheral colours that blended smoothly with the intact central region (Figure 8), demonstrating that the problem is, at least in principle, solvable from the statistical regularities of natural environments. Crucially, the DNN could not have simply learned to increase saturation in the periphery, because at the greatest eccentricity colour was completely removed.

Given the high realism of the network output, we do not claim that the only mechanism involved in the machine learning was the memory colour effect. In fact, the network may have learned to extrapolate central colours to the periphery of objects effectively increasing the changes to reproduce the ground truth. Although such models are not intended as direct models of human vision, they illustrate that the information required to infer plausible peripheral colour is present in natural scenes, lending computational support to the hypothesis that the visual system could exploit similar regularities to maintain the vivid appearance of colour across the visual field.

**Figure 9:**
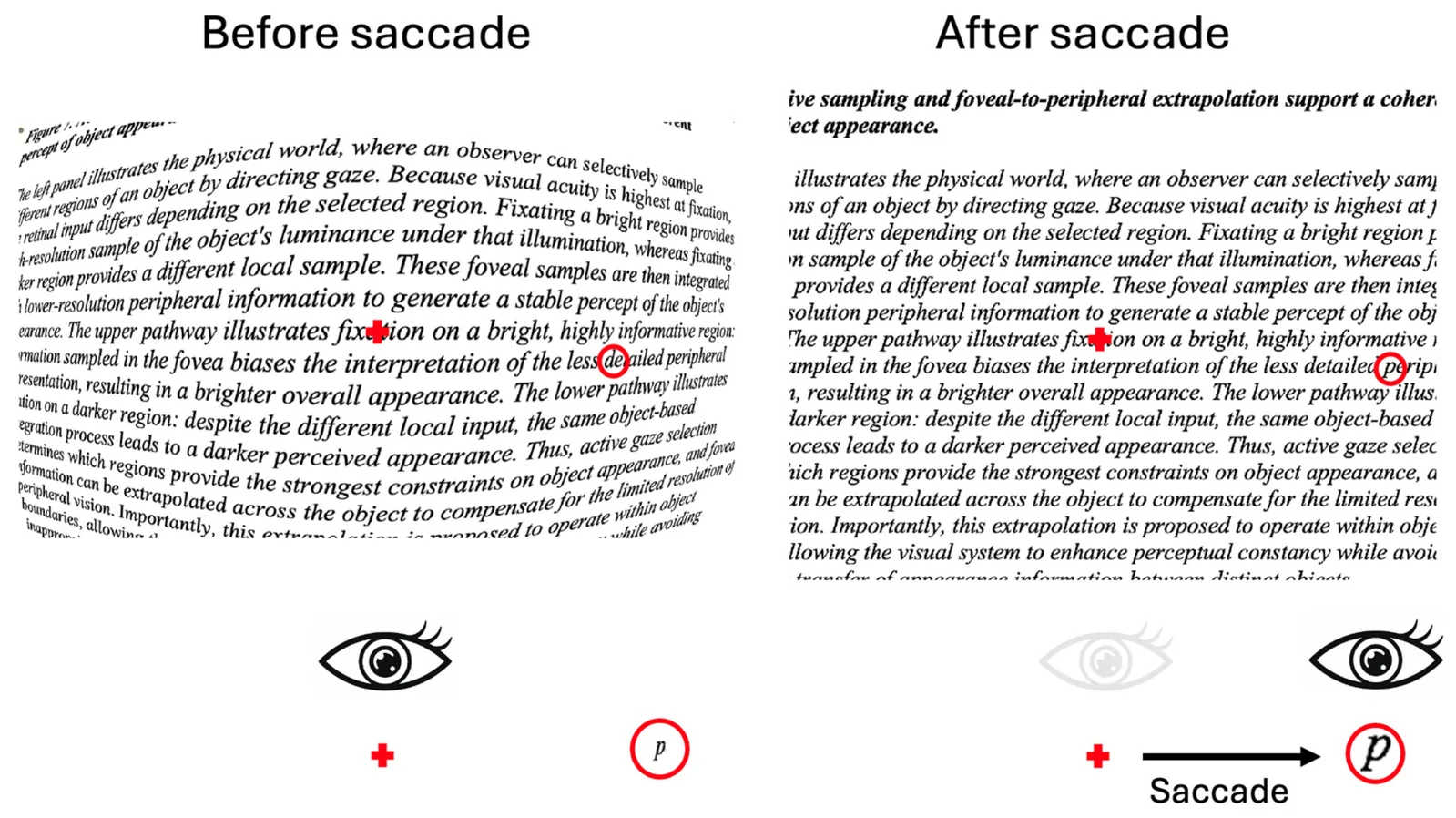
Recalibration: learning how a peripheral object will appear when fixated. Size is distorted in peripheral vision, as illustrated in the left image. The cross indicates the fixation point, and the circles highlight a peripheral element—the letter p—which appears larger than the letters closer to fixation. The right image shows the original, undistorted scene. After a saccade, the letter p is brought to fixation and is perceived without the distortions associated with peripheral vision. Through repeated eye movements, the visual system may learn the correspondence between an object’s peripheral appearance and its foveal appearance, allowing it to recalibrate peripheral perception accordingly.

While statistical regularities can be exploited, another source of information that the visual system can learn through eye movements is the correspondence between how an object appears in peripheral vision and how it appears after a saccade brings it into central vision. Through repeated visual experience, the system can learn the relationship between the lower-resolution peripheral representation and the more detailed foveal representation that follows fixation. This learned mapping could provide a powerful source of information for interpreting peripheral input, allowing peripheral vision to be enriched by predictions based on prior experience with the same objects viewed centrally (Figure 9).

Such learning has been demonstrated for size perception [59,95]. Controlled perceptual matching experiments show that, to appear at the same spatial frequency as a centrally viewed stimulus, observers adjust the spatial frequency of sinusoidal gratings presented in the periphery to be lower, indicating that spatial frequency is perceived as higher in peripheral vision [17,96]. Similarly, in most experiments, the size of a peripheral object or patch needs to be increased to appear equivalent in size to a centrally viewed one, indicating that perceived size is compressed in peripheral vision [97,98,99]. This makes size perception a suitable candidate for testing whether the visual system compensates for such distortions based on learned correspondences between how an object appears in peripheral vision and how it appears after a saccade when brought into central vision.

Valsecchi and Gegenfurtner (2016) [59] presented two circular stimuli, one centrally and one peripherally, and asked participants to compare their perceived sizes. This allowed them to estimate the perceived size of the peripheral stimulus. After the size-comparison task, participants were instructed to make a saccade towards the peripheral stimulus. Crucially, during the saccade, when perception is suppressed [43] and participants did not notice, the size of the stimulus was either decreased or increased by 10% in different blocked conditions. This trans-saccadic manipulation induced a corresponding change in the perceived size of the peripheral stimulus relative to the foveal stimulus, demonstrating that participants learned the correspondence between how the stimulus appears in peripheral vision and how it will appear once fixated.

Importantly, the recalibration occurred rapidly: observers did not need extensive exposure over days or weeks but adapted within the experimental session. This suggests that the visual system continuously updates the relationship between peripheral and foveal representations based on the consequences of eye movements.

The temporal and spatial properties of this recalibration process were subsequently investigated by Valsecchi and colleagues [100], who compared trans-saccadic perceptual recalibration with the better-established phenomenon of saccadic adaptation. Although both processes are driven by systematic discrepancies between predicted and actual outcomes of eye movements, they involve different aspects of behaviour: saccadic adaptation modifies the motor command controlling eye movements, whereas perceptual recalibration modifies the perceived appearance of objects. To characterise the dynamics of perceptual recalibration, Valsecchi et al. introduced controlled oscillations in the size changes occurring during the saccade. Instead of applying a constant increase or decrease in stimulus size, as in their previous work, the magnitude of the trans-saccadic size change varied sinusoidally across trials. If observers continuously updated their estimate of peripheral size based on the post-saccadic error, their perceived size should track these oscillations with some delay. This was indeed observed: changes in perceived peripheral size followed the imposed trans-saccadic changes, with a lag of fewer than ten trials. This rapid adjustment indicates that the visual system can update the mapping between peripheral and foveal appearance dynamically, rather than relying on a slow learning process requiring extensive exposure.

The authors further examined whether perceptual recalibration is spatially specific. This was motivated by comparisons with saccadic adaptation, where learning is typically restricted to the particular saccade direction and target location that generated the error [101,102,103,104]. In contrast, size recalibration showed almost complete generalisation to the corresponding location in the opposite visual hemifield. This difference suggests that perceptual recalibration does not simply adjust a local sensorimotor transformation associated with a specific eye movement. Instead, it appears to update a more global representation of how objects are expected to appear across the visual field.

Such learning has been demonstrated not only for size perception but also for more complex properties of object appearance. Paeye and colleagues (2018) [105], investigated whether the visual system can learn and compensate for systematic distortions in peripheral shape perception. Participants were exposed to objects whose shape was systematically changed as they transitioned from peripheral to foveal vision, either during a saccade or during a controlled displacement without an eye movement. Specifically, objects changed from more circular to more triangular shapes, or vice versa, between peripheral and central viewing. In a subsequent test phase, observers’ perceived shape was biased according to this previous experience: objects were perceived as less curved when they had previously changed from a more circular peripheral appearance to a more triangular foveal appearance, and as more curved when they had previously changed in the opposite direction. Importantly, this recalibration occurred both with and without saccadic eye movements, demonstrating that it does not depend on the motor act of making a saccade but reflects a more general process through which the visual system learns correspondences between different visual representations. Further evidence for a predictive contribution to peripheral appearance comes from Valsecchi and colleagues (2018) [106], who showed that observers’ perception of peripheral images is influenced by assumptions about how those images would appear in central vision. Using controlled distortions of geometric shapes and natural images, they found that observers tended to perceive peripheral stimuli as less distorted and more similar to their expected foveal appearance than the physical stimulus actually was, indicating that peripheral perception is complemented by predictions about future sensory input.

## 5. Open Questions

While recent research suggests that the perceived richness of peripheral vision and perceptual uniformity are unlikely to be simply a product of cognitive bias, but instead reflect genuine perceptual phenomena, and has highlighted potential mechanisms supporting this experience, we are still far from a comprehensive theory of how peripheral vision gives rise to a rich, stable, and coherent perceptual experience despite the severe limitations of peripheral sensory input.

**Figure 10:**
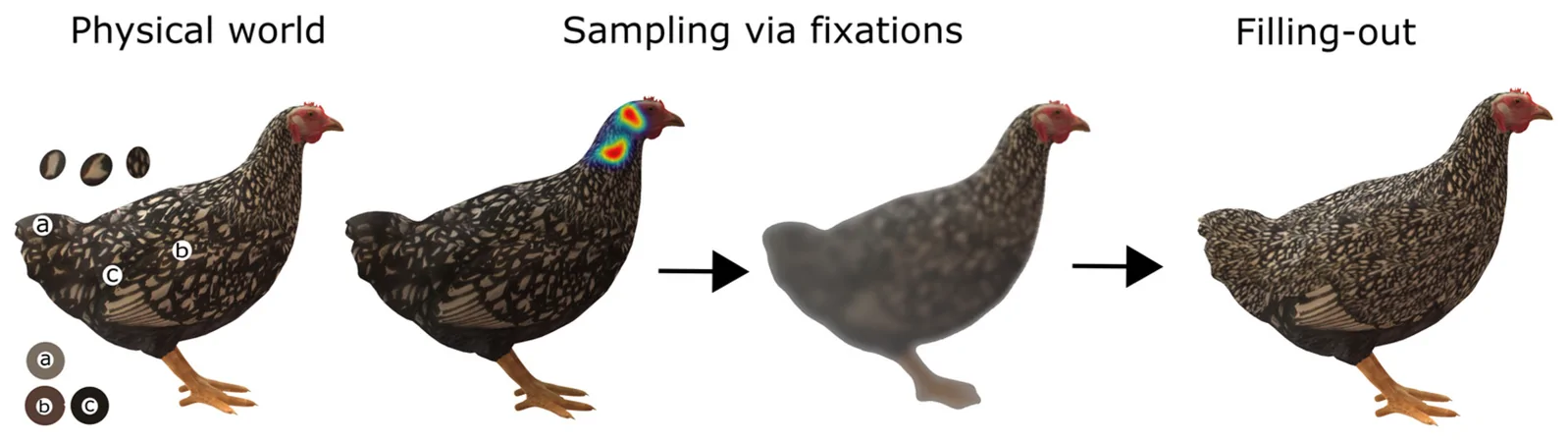
Active perception and filling-out. Physical objects exhibit variability in their visual properties, such as the shape and size of individual elements (e.g., the speckles, which are larger in the hen’s tail, as shown in the images above), as well as variations in colour and luminance, as illustrated in (a), (b) and (c). However, when we look at an object, we do not exhaustively sample its entire surface and store a collection of high-resolution snapshots that are later combined. Instead, we selectively sample the most informative regions, such as the head of the hen in this example, as shown by the simulated heat map representing fixation density, while leaving the remaining areas in peripheral vision. Filling-out mechanisms then extrapolate information from the fixated regions to areas that are only coarsely represented in peripheral vision. This can be seen in the smaller speckles in the hen’s tail on the right, which resemble the size of those near the head, where fixations are more likely to land.

A working framework is that vision is an active process in which observers continuously sample the world through eye movements, selectively bringing task-relevant objects and features into foveal vision. Eye movements allow observers to acquire information from diagnostic regions that are particularly informative for recognising objects and guiding behaviour. Importantly, information extracted from these diagnostic regions can influence the appearance of the whole object, including regions that remain poorly represented in peripheral vision. For example, when judging the lightness of an object, the visual system may not estimate the lightness of every region independently based on degraded peripheral signals. Instead, information from more reliable regions of the object may be used to infer a coherent surface appearance, influencing the perceived lightness of the object as a whole through perceptual filling-out. Similar processes may operate for other properties, including colour, texture, shape, and material appearance (Figure 10). However, filling-out cannot operate indiscriminately across space. If information from one object were freely extrapolated to another, it would generate confusion and produce incorrect interpretations of the visual scene. Therefore, additional mechanisms must constrain where and how information is extrapolated (filling-out). These constraints could arise from object segmentation, prior knowledge about objects, and learned expectations about how objects will appear under different viewing conditions. In this sense, perceptual filling-out is unlikely to be a purely local interpolation process; instead, it may interact with higher-level representations that determine which information belongs together and how it should be integrated.

Several fundamental questions remain unresolved. First, it is not known which features are affected by filling-out or whether different features rely on different mechanisms. Filling-out may operate through relatively low-level mechanisms for some properties, such as luminance or colour, but involve higher-level representations for more complex properties, such as shape, object identity, or material appearance. The neural correlates of filling-out (specifically of the uniformity illusion) have been investigated only indirectly [65,71], and the relationship between neural responses associated with perceptual completion and the perceptual experience itself remains unclear. Importantly, differences in neural implementation between features may provide critical information about the mechanisms involved, i.e., whether reconstruction occurs at early sensory stages or depends on higher-level object representations.

**Figure 11:**
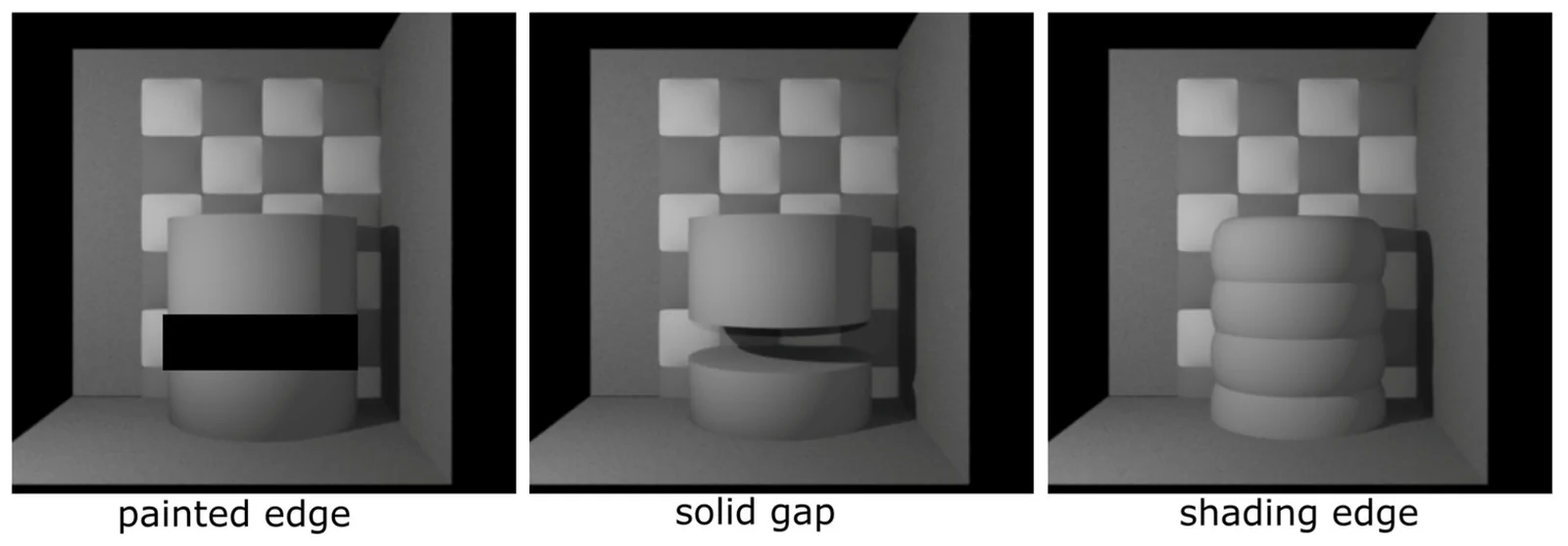
Different types of edges can influence perceptual filling-out in different ways.

A related unresolved question concerns the nature of the boundaries that constrain perceptual extrapolation. According to a purely low-level account, the effects of different types of borders should be comparable as long as their low-level image properties, such as contrast and spatial frequency, are matched. Under this account, any sufficiently strong edge should stop filling-out, regardless of whether it represents a luminance change, a shading transition, or an object contour (Figure 11). However, if object segmentation contributes to the process, the visual system should distinguish between different types of boundaries. For example, a shading edge may indicate a change in illumination or surface orientation while preserving object continuity, whereas a solid gap or occluding contour defines a separation between objects and should prevent information from one object influencing another. Determining whether filling-out is constrained by physical edges or by object boundaries therefore provides a direct test of the level at which perceptual reconstruction occurs.

This possibility that object segmentation is involved is supported by evidence that the visual system retains substantial object-level processing capabilities in peripheral vision. Humans can rapidly detect and categorise objects in briefly presented natural images [107], suggesting that peripheral vision is not limited to a purely low-level representation but retains access to higher-level information relevant for object recognition. Such object-level processing could provide the segmentation and contextual information necessary to constrain filling-out and determine which information should be extrapolated and which should remain separate.

An important complementary perspective comes from the Texture Tiling Model of peripheral vision [54,108], which proposes that peripheral input is represented not as a collection of individually specified elements but in terms of summary statistics computed over pooling regions that increase in size with eccentricity. On this account, information about the distribution and correlations of features can be retained while their precise spatial arrangement and object identity are lost or degraded. This raises an important qualification for the idea of peripheral filling-out: filling-out need not involve the extrapolation of each individual element or feature from central vision. Instead, what is extrapolated or inferred may be a higher-order statistical description of the scene, preserving properties such as texture, feature distributions, and regularities while sacrificing precise local detail. In this sense, summary-statistic and object-based accounts need not be mutually exclusive. Object and scene knowledge could constrain the statistical representation generated in the periphery, while the statistical representation could determine the level of detail at which that extrapolated content is experienced. The phenomenological richness of peripheral vision might therefore arise from structured, statistically constrained extrapolation rather than from a literal reconstruction of individual objects and features.

Whether, and under what circumstances, individual peripheral elements are extrapolated from foveal information, or whether summary-statistic representations are instead propagated from the fovea to the periphery, remains an open empirical question. Nevertheless, the uniformity illusion demonstrates that peripheral filling-out can occur for textures [56], and our own work suggests that similar effects extend to complex ensembles such as crowds of faces [57]. Such ensembles are known to be represented in terms of summary statistics, with observers able to extract properties such as the mean identity or emotional expression of a crowd without necessarily individuating its members [78]. These findings raise the possibility that what is experienced as rich peripheral content may in some cases reflect the propagation or construction of ensemble-level representations rather than the reconstruction of individual elements. This possibility may also help explain why the orientation aftereffect following the uniformity illusion was determined by the physical, rather than the illusory, orientation of individual peripheral elements [65], whereas aftereffects have been demonstrated for colour and size [71], suggesting that the extent to which the illusory peripheral representation is instantiated at the level of local features may depend on the feature or on the level of representation involved.

An important consideration is the relatively slow time course of the classic uniformity illusion. The illusion typically develops only after several seconds of sustained fixation [56], which is considerably longer than the duration of a typical fixation during natural viewing. However, this may not necessarily reflect the time required for centre-to-periphery propagation itself. The classic uniformity illusion displays contain a strong discontinuity between the central and peripheral regions, which may function perceptually as a boundary between two regions with different visual properties. In our work on lightness extrapolation [32], we found that the propagation of central information into the periphery was constrained by object boundaries, suggesting that such boundaries play an important role in determining where central information is extended. The relatively slow development of the classic uniformity illusion may therefore partly reflect the need to overcome or reinterpret the strong centre–periphery discontinuity, rather than the intrinsic temporal dynamics of centre-to-periphery propagation. Preliminary evidence for this comes from a version of the uniformity illusion presented at ECVP 2026 (Authors: Valsecchi, Sharvashidze, & Toscani), in which there was no explicit centre–periphery boundary: dots in the centre were either connected by lines or unconnected, whereas the peripheral dots were not connected. Despite the absence of an explicit boundary, participants experienced the central connectivity property as extending into the periphery following only 1 s of stimulus presentation. A further possibility is that the relatively slow development of the classic uniformity illusion partly reflects the time required for the conflicting peripheral signal to weaken under sustained fixation. Stationary peripheral stimuli are known to undergo Troxler fading, gradually becoming less visible; depending on stimulus conditions, this can take several seconds or longer (e.g., [109,110]). Notably, texture-fading studies suggest that an explicit boundary between a peripheral target and its surround can itself influence the time course of fading, with fading becoming faster when the abutting contour is removed [111]. Thus, in the classic uniformity illusion displays, the strong discontinuity between central and peripheral textures may both impede centre-to-periphery propagation and delay the fading or suppression of the conflicting peripheral information. Moreover, the relevant timescale during natural vision need not be limited to the duration of a single fixation as in naturalistic, goal-directed behaviour, gaze is closely coupled to task-relevant objects which observers may repeatedly or continuously sample over the course of an action [20].

Beyond filling-out, memory and prior knowledge represent another potential source of information supporting peripheral perception. Similar to filling-out, memory can compensate for unreliable sensory input, but it remains unclear which features can be reconstructed from experience. Does the visual system use prior knowledge only to complete low properties such as colour or lightness, or can it influence complex aspects of appearance, such as shape, material, or object identity?

The role of attention represents another major unresolved issue. The apparent richness of peripheral vision may depend strongly on attentional selection [42,43,112]. Using immersive virtual reality and gaze-contingent rendering, Cohen et al. (2020) [113] demonstrated that observers are often unaware of dramatic reductions in peripheral visual information during naturalistic viewing. Participants explored dynamic real-world environments while only the region surrounding their current fixation remained in colour, with the remainder of the visual field progressively desaturated. Despite these extensive alterations, observers frequently failed to notice that large portions of their visual world had become colourless, with some participants remaining unaware even when less than 5% of the display was presented in colour. Is peripheral information continuously reconstructed through filling-out and prior knowledge, or are these mechanisms engaged only when information becomes behaviourally relevant?

One possibility is that the visual system does not need to represent the entire visual field in detail at any given moment. This idea is central to theories of active vision. Rensink (2000) [114] proposed that, rather than maintaining a detailed, continuously updated representation of the entire scene, vision constructs relatively stable representations of objects and regions when they are selected by attention. In this view, the apparent continuity of the visual world does not require all of its contents to be simultaneously represented in detail; instead, attention dynamically determines which aspects of the scene are represented and maintained. Similarly, Ballard and colleagues proposed that visual information is acquired strategically in relation to the current task and action [115,116,117]. In their “just-in-time” account [23], observers acquire information when it becomes relevant, with eye movements bringing relevant objects into high-resolution vision, rather than maintaining detailed representations of everything that might eventually be needed.

This possibility is particularly relevant to peripheral vision. The visual system may not need to enrich or fill in every portion of the peripheral visual field, but only those portions that are relevant to the current task. Covert attention could select a peripheral object or region before it is brought to fixation, allowing perceptual mechanisms such as filling-out or the use of prior knowledge to contribute selectively to its representation. Information about a task-relevant object could therefore be elaborated while it remains in the periphery, potentially providing a sufficiently stable representation to guide subsequent behaviour and maintain perceptual continuity when the object is fixated. By contrast, peripheral information that is neither attended nor relevant to the current task may remain relatively coarse and need not undergo the same degree of perceptual elaboration.

Our studies of lightness perception provide a concrete example of this “just-in-time” task-dependent selection. We found that observers preferentially fixated regions of an object that were informative for judging its lightness, and that manipulating which regions were fixated systematically affected the perceived lightness of the object [26,27]. In a subsequent study using a moving stimulus, we found that lightness judgments were determined predominantly by the visual information sampled around the time at which the judgment had to be made [33]. Information sampled earlier in the viewing period contributed substantially less or not at all to the final judgment, indicating that observers did not simply accumulate and equally weight all information sampled over time. Instead, information available around the moment of judgment was given greater weight, whereas information sampled earlier was largely discounted. Even when information has previously been sampled, its influence on perception depends on its relevance to the observer’s current perceptual goal.

From this perspective, active vision and perceptual filling-out may be closely intertwined. Peripheral information can identify objects and regions that are relevant to the current task; covert attention can select those regions; and filling-out and prior-based inference could then contribute to their perceptual representation before subsequent foveal sampling. The remainder of the visual field may require no such detailed reconstruction and can remain relatively coarse or largely unnoticed until it becomes behaviourally relevant. The apparent richness of peripheral experience could therefore emerge from a temporally extended interaction between peripheral information, attentional selection, perceptual inference, and active sampling, rather than from a requirement that the entire visual field be represented in high detail simultaneously.

Interestingly, this perspective may also provide a way of reconsidering the inflation account discussed earlier. Earlier work showed that observers can adopt more liberal detection criteria for unattended stimuli even when perceptual sensitivity is matched across conditions [118]. More directly, Solovey et al. (2015) [119] found a more liberal detection criterion for peripheral than for central stimuli when detection sensitivity was matched and explicitly hypothesised that the same mechanism proposed for unattended vision by Rahnev et al. (2011) [118] may operate in the visual periphery. Similar effects have been reported in more naturalistic settings: during simulated driving, observers showed liberal detection biases when judging the colour of pedestrians’ clothing at unattended peripheral locations [120]. These findings provide converging evidence that peripheral vision can be associated with systematic biases in subjective reports or decision criteria. A computational account of this relationship was proposed by Winter and Peters (2022) [121], who modelled peripheral inflation as arising from inaccurate assumptions about the statistics of sensory noise. Their simulations showed that inflation, and particularly its increase with reduced peripheral attention, could emerge when the observer incorrectly estimates the distribution of noise affecting peripheral representations.

Considered together with attention and active vision, these findings suggest a possible reconciliation between inflation and the constructive mechanisms discussed in this review. Inflation need not apply uniformly across peripheral vision. Instead, it may be particularly relevant to the large portion of the visual field that is not currently selected by attention. Task-relevant peripheral information can be selected covertly and may subsequently be elaborated through interactions with foveal information, prior knowledge, and perceptual inference, whereas unattended peripheral information may remain comparatively coarse or uncertain. Inflation could contribute to why this unattended portion of the visual field nevertheless does not appear correspondingly impoverished.

Such an account is consistent with Rensink’s (2000) [114] proposal that a coherent visual experience does not require a detailed representation of all objects in a scene simultaneously. As attention and gaze move through the environment, different portions of the visual field can become selected and elaborated as they become relevant. Apparent perceptual richness may therefore arise from a combination of mechanisms operating on different information at different moments: selective perceptual construction for attended and task-relevant information, together with possible inflation of the much larger amount of information that remains unattended.

Overall, current evidence suggests that peripheral perception relies on multiple interacting mechanisms. Filling-out may allow information from reliable regions to constrain the appearance of less reliable regions, but it may operate only within object boundaries when part of the object is at or near fixation. Learned predictions based on experience may enable the visual system to anticipate how objects will appear following changes in viewpoint or retinal location, while prior knowledge may help fill in missing information about objects in peripheral vision. However, it remains unclear whether these mechanisms are sufficient or whether additional processes contribute to the richness and stability of peripheral perception. A major limitation of current research is that many classical studies of peripheral vision rely on simplified stimuli, such as isolated gratings or geometric shapes, which may underestimate the contribution of contextual information and object knowledge.

**Figure 12:**
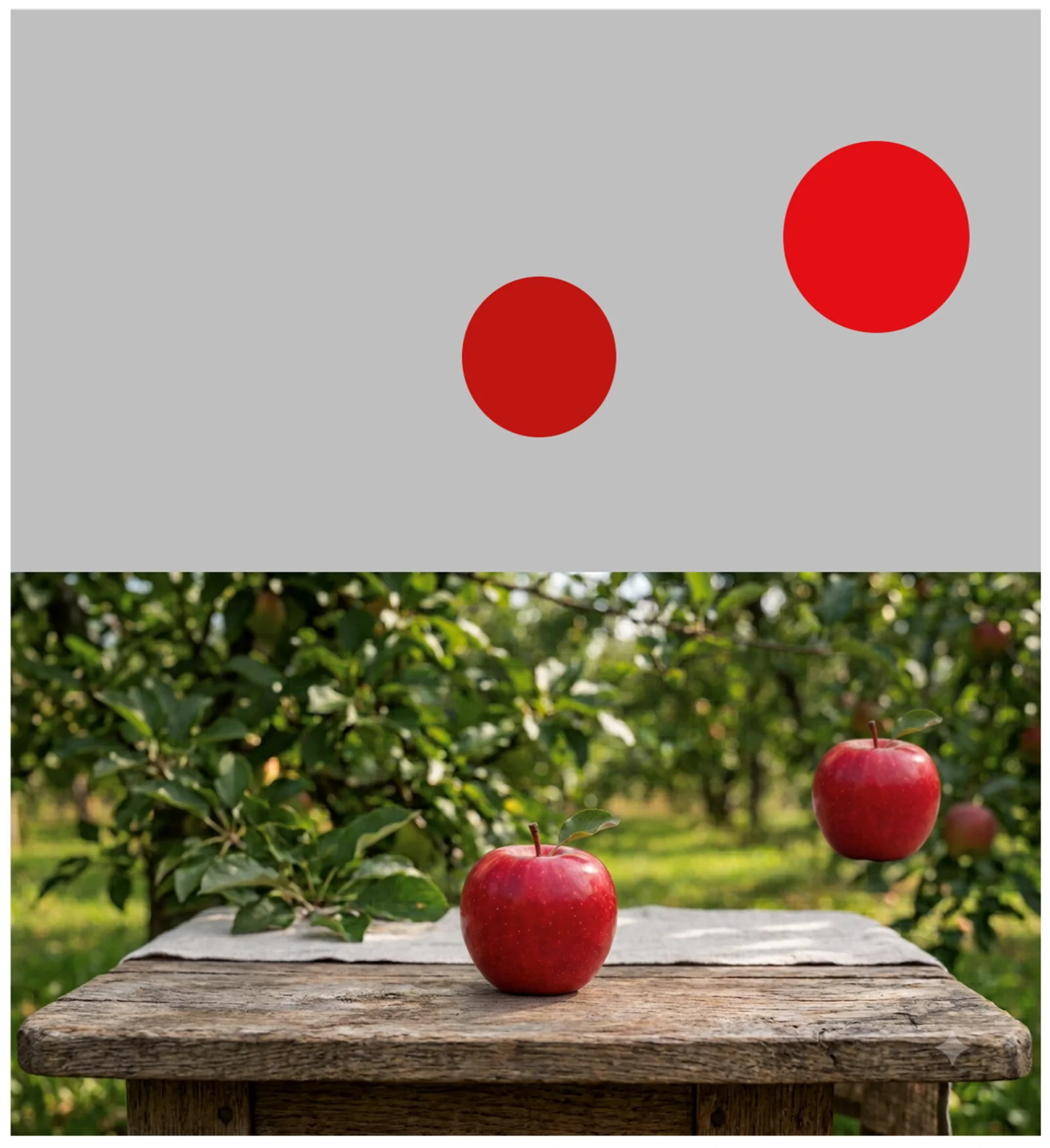
In a classic peripheral vision study (**top**), properties of the peripheral stimulus are adjusted to match the appearance of the central stimulus. In this example, the matching procedure resulted in a brighter and larger match, to compensate for smaller size and darker brightness biases. However, in natural conditions (**bottom**), the biases can be compensated by contextual information.

For example, measuring perceived size or colour using isolated circles (Figure 12) presented centrally and peripherally may reveal strong perceptual distortions, but these distortions may be reduced, transformed, or compensated when the same information is embedded within meaningful objects and scenes.

Natural objects provide additional constraints through boundaries, context, category knowledge, and expectations. Revisiting classical peripheral vision phenomena using ecologically valid stimuli could therefore reveal which biases represent genuine limitations of peripheral representations and which are normally compensated by higher-level mechanisms. Rather than asking only how the visual system corrects peripheral distortions, this approach would help determine how perception emerges from the interaction between sensory limitations, active sampling, perceptual filling-out, attention, memory, and learned predictions.

## Figures and Tables

**Table 1:** Different accounts for rich visual experience in peripheral vision.

Account	Core Proposal	Key Evidence
**Metacognitive inflation**	Observers overestimate the amount of peripheral detail available to them.	Confidence–performance dissociations; overestimation of peripheral detail (e.g., Odegaard et al., 2018 [51]).
**Summary-statistics/metamer accounts**	Peripheral information is compressed into higher-order statistics, sacrificing precise spatial arrangement and individual elements.	Texture tiling (Rosenholtz et al., 2012 [54]); metamers (Freeman & Simoncelli, 2011 [55]).
**Filling-out/extrapolation**	Information sampled centrally can influence and be extended into peripheral representations.	Uniformity illusion (Otten et al., 2017 [56]); lightness and other feature-propagation effects (Toscani et al., 2017 [32]; Zoghlami et al., 2022 [57]).
**Predictive processing/Bayesian inference**	Perception combines noisy sensory evidence with prior knowledge of object properties and perceptual experience and learned environmental regularities.	Predictive-processing and Bayesian models of perception. Particularly strong effects of prior knowledge for peripheral colour perception [58]. Dynamic recalibration of a learned contingency between peripheral and foveal representations of the same object (e.g., Valsecchi and Gegenfurtner, 2016 [59]).

## Data Availability

No new data were created or analysed in this study. Data sharing is not applicable to this article.

## Abbreviation

The following abbreviation is used in this manuscript:

DNN	Deep Neural Network
